# Heme sequestration and signaling in molecular condensates

**DOI:** 10.1039/d6sc06692e

**Published:** 2026-10-22

**Authors:** Ananya Mishra, Andrea E. Gallio, Emma L. Raven, Avinash J. Patil, Stephen Mann

**Affiliations:** a Centre for Protolife Research and Centre for Organized Matter Chemistry, School of Chemistry, University of Bristol Bristol BS8 1TS UK s.mann@bristol.ac.uk; b School of Chemistry, University of Bristol Bristol BS8 1TS UK; c Max Planck-Bristol Centre for Minimal Biology, School of Chemistry, University of Bristol Bristol BS8 1TS UK

## Abstract

Understanding the mechanisms responsible for intracellular heme regulation remains a significant challenge. Here, we use molecularly crowded condensate droplets to sequester heme-binding sensor proteins that are subsequently activated by the uptake of free heme from aqueous solution. We demonstrate competitive heme binding and heme-mediated signaling within the membraneless coacervate droplets, and construct programmable synthetic/living cell communication networks capable of concatenated Boolean logic functions. Our results indicate that coacervate microdroplets are potential scaffolds for housing heme-sensor networks and provide a simplified model for exploring how biomolecular condensates may influence heme availability in cellular environments.

## Abstract 2

Uptake of free heme into molecularly crowded coacervate microdroplets activates encapsulated sensor proteins and provides a step to heme regulation, signaling and biomolecular computation in synthetic/living condensate networks.

## Graphical abstract

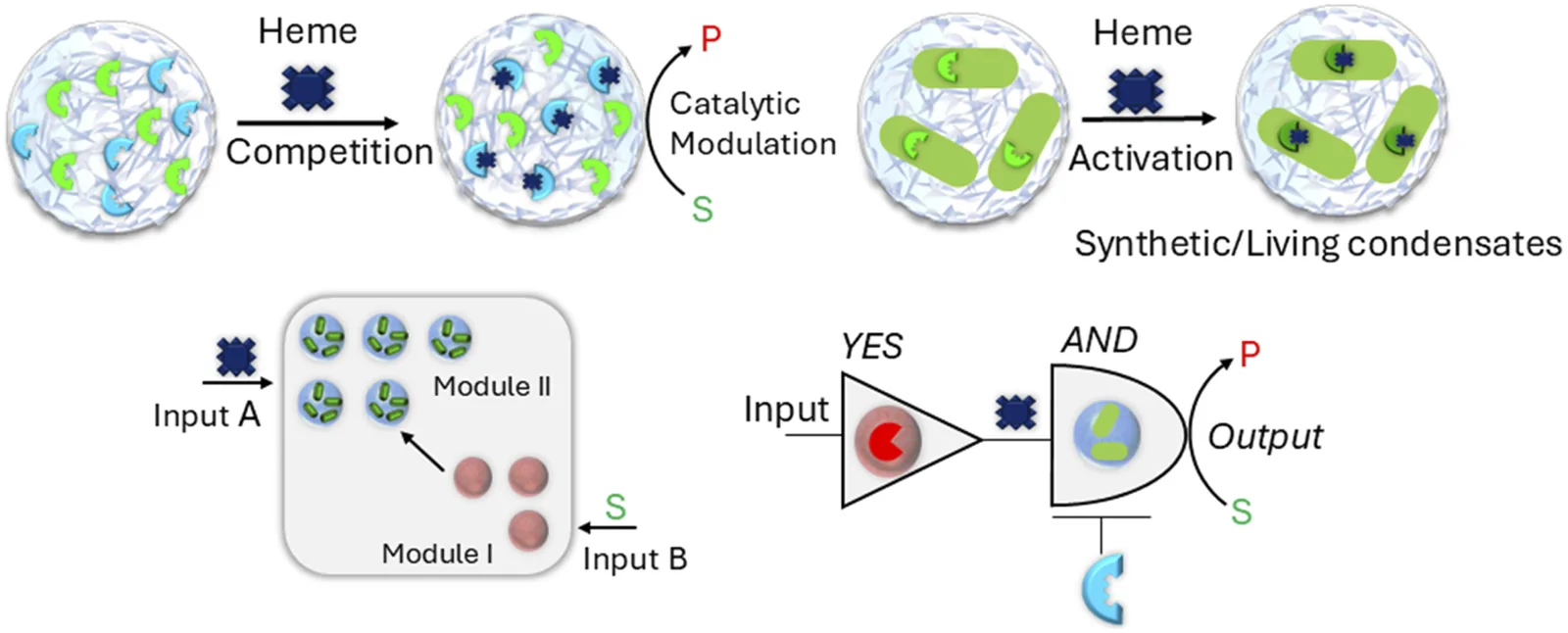

## Introduction

The iron protoporphyrin IX (heme) complex is an essential biological cofactor used in oxygen storage and transfer, electron shuttling and redox-coupled reactions.^1–3^ Reversible binding of heme to a range of proteins facilitates intracellular signalling, protein assembly, regulation of gene expression and ion channel functions.^4,5^ Recent studies have explored the role of heme in synthetic cellular systems, investigated heme transport across lipid membranes,^6^ heme-triggered catalysis,^7^ and the activity of hemin in non-water based solvents.^8^ These studies indicate that the distribution of heme can be regulated by the thermodynamically driven transfer of heme specifically from low- to high-affinity heme-binding proteins.^9–11^ Moreover, while the reactivity of free heme in aqueous solution is low due to minimal solubility, the diverse range of protein binding affinities allows for control over heme-mediated catalysis,^12–16^ and the regulation of heme homeostasis, thereby mitigating the accumulation of free potentially toxic heme and oxidative stress.^16–19^

Replicating hierarchical mechanisms that control heme availability within synthetic systems offers a promising strategy for designing controlled and functional biomimetic environments.^20^ As heme availability and inter-protein heme exchange occur within molecularly crowded intracellular environments, we speculate that biomolecular condensates may contribute to heme homeostasis by reversibly sequestering and exchanging heme on demand. To explore this possibility, here we use molecular condensates in the form of membraneless, molecularly crowded polymer-nucleotide coacervate microdroplets containing heme-binding sensor proteins that are activated by the uptake of free heme from aqueous solution. We demonstrate that the coacervate droplets can spontaneously sequester heme-binding proteins, enabling the real-time observation of heme binding, inter-protein heme exchange, and heme-based signalling within the condensates. We investigate heme regulation through competitive binding among proteins with different heme-binding affinities and examine the impact on their catalytic activity within molecularly crowded environments. Given that coacervate phases facilitate biocatalysis^21^ and support communities of living cells,^22–25^ we use heme-mediated chemical signaling to construct synthetic/living cell communication networks in molecular condensate droplet arrays. Our approach provides a step to switchable biochemical functions and communication circuits in networks of compartmentalized enzymes, offers opportunities for the construction of heme-based micro-sensor arrays, and supports a biomolecular condensate model of intracellular heme management.

## Results and discussion

### Compartmentalised heme sequestration and biocatalytic activation in acoustically patterned coacervates

Acoustically patterned 2D arrays of protein-containing poly-diallyldimethylammonium chloride (PDDA)/adenosine triphosphate (ATP) coacervate microdroplets typically 95 µm in mean size (Fig. S1) and organized with a centre-to-centre lattice spacing of 107 ± 7 µm were prepared by using an in-house acoustic trapping device comprising two orthogonally placed pairs of transducers operated at 6.7/6.69 MHz (Fig. 1a). The arrays were employed as a platform for the heme-triggered activation and logical computation of spatially distributed embodied enzyme networks. The uptake of heme was monitored by time-dependent quenching of the fluorescence intensity of an intra-droplet heme-binding protein (monomeric ascorbate peroxidase, mAPX, in the heme free, *apo*, form) fused to a monomeric green fluorescent protein (mEGFP), *apo*-mAPXmEGFP.^26,27^ The resulting peroxidase activity of the heme-bound *apo*-mAPXmEGFP protein (*holo*-mAPXmEGFP) within the molecular condensates was determined using an Amplex red/hydrogen peroxide (H_2_O_2_) assay. A six-fold molar excess of hemin (30 µM) was added to *apo*-mAPXmEGFP to ensure saturation in protein binding as observed from titration experiments (Fig. S2).

To assess whether low concentrations of aqueous heme could be used as an input for the activation of enzyme reactivity in the PDDA/ATP coacervate microdroplets, we determined the heme equilibrium partition constant (*K*) in bulk coacervates prepared in the absence of *apo*-mAPXmEGFP at pH 8 using UV-vis spectroscopy. The measurements (*K* = 5.6) indicated that the coacervate microdroplets provided an effective sink for the accumulation of heme from dilute aqueous solution. Typically, the intra-droplet heme concentration was increased to *ca*. 160 µM when incubated in a 30 µM saturated solution of heme. The spontaneous sequestration was attributed to favourable non-covalent interactions between heme and the molecularly crowded partially apolar environment of the PDDA/ATP matrix.^28–31^

**Fig. 1:**
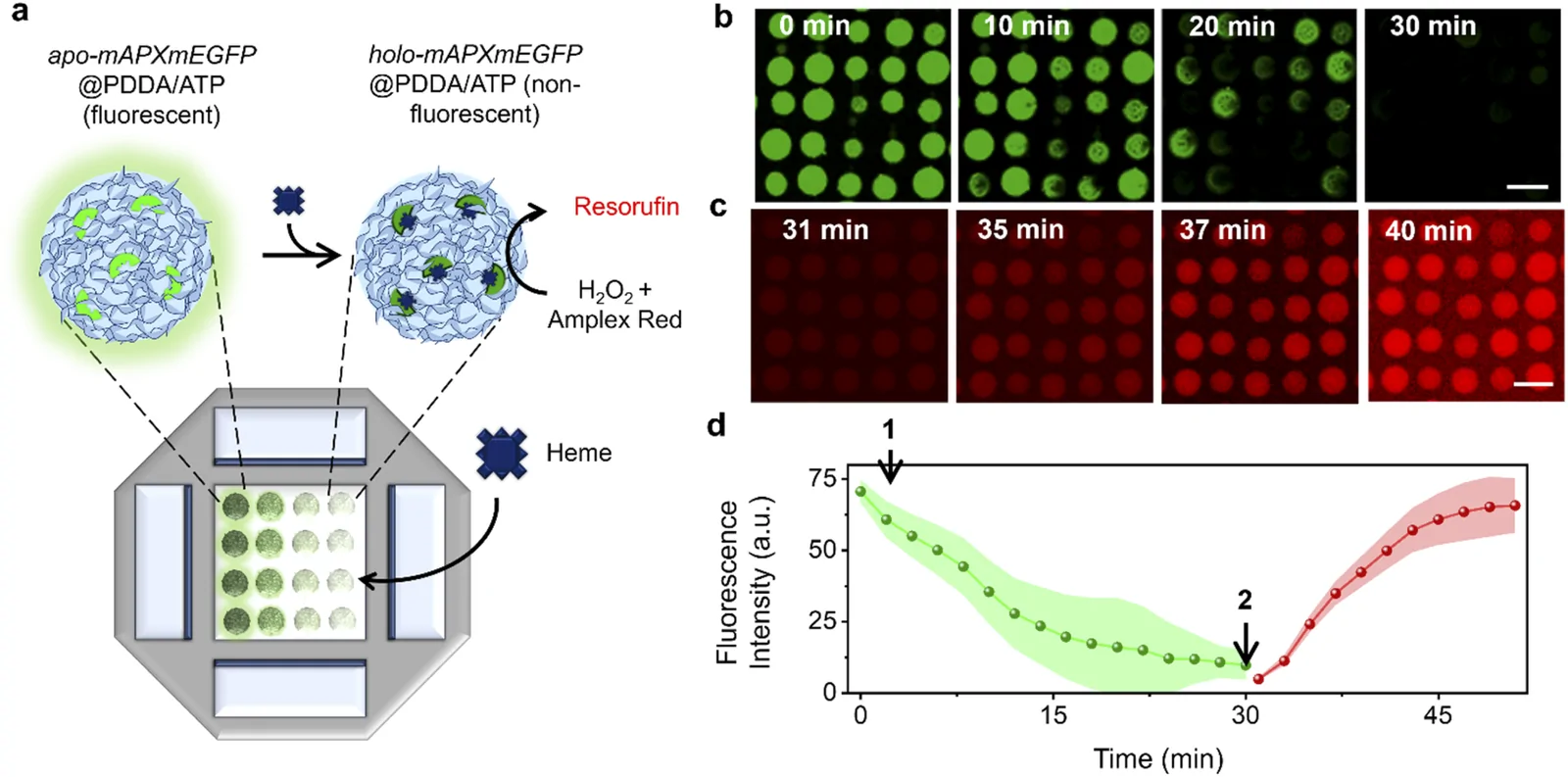
Heme sequestration and biocatalytic activation in molecular condensates. (a) Scheme illustrating acoustic patterning of a 2D microarray of *apo*-mAPXmEGFP-encapsulated PDDA/ATP coacervate droplets. Injection of aqueous heme (dark blue symbol) into the sample chamber gives rise to partitioning of heme into the condensate droplets. Subsequent binding to *apo*-mAPXmEGFP quenches the green fluorescence and produces *holo*-mAPXmEGFP, which exhibits peroxidase activity in the presence of Amplex red and H_2_O_2_ to generate a red fluorescent output (resorufin). (b and c) Time series of fluorescence microscopy images showing quenching of intra-droplet *apo*-mAPXmEGFP (5 µM) green fluorescence after addition of heme (30 µM, *t* = 0) (b) and increase in intra-droplet red fluorescence (resorufin production) after conversion to *holo*-mAPXmEGFP and addition of Amplex red and H_2_O_2_ (*t* = 31 min) (c). Scale bars, 100 µm. (d) Plot of time-dependent changes in fluorescence intensity recorded from samples shown in (b) and (c). Time points for the addition of heme (*t* = 0 min, arrow 1) and H_2_O_2_/Amplex red (*t* = 31 min, arrow 2). Green and red fluorescence intensity measurements (a.u.) are collected from 5 × 5 grids of coacervate microdroplets from the same experiment but different fields of view; shaded error regions are obtained from triplicate experiments.

Fluorescence microscopy images of 2D arrays of PDDA/ATP coacervate microdroplets containing *apo*-mAPXmEGFP at pH 8 (*K* = 2.7) showed a homogenous distribution of green fluorescence within the molecular condensate, confirming sequestration of the heme-sensor fusion protein (Fig. 1b). Addition of an aqueous solution of heme to the sample chamber progressively quenched the *apo*-mAPXmEGFP green fluorescence over a period of *ca*. 30 min (Fig. 1b), consistent with heme uptake, binding to the incarcerated reporter protein, and onset of Förster resonance energy transfer (FRET) within the *holo*-mAPXmEGFP complex (Fig. S3 and S4).^27^ To investigate heme-induced activation of mAPx within the coacervate droplets, we monitored the peroxidase-like activity of encapsulated *holo*-mAPXmEGFP by introducing H_2_O_2_ and Amplex Red to both the acoustically patterned 2D microarray monitored at pH 8 and coacervate microdroplets prepared in the absence of an acoustic field at pH 7 (Fig. S5–S8 and Table S1). In the latter case, the variation in heme uptake and biocatalytic activity were possibly associated with the polydisperse size distribution and the resulting differences in substrate diffusion. In contrast, time-lapse fluorescence microscopy images of 2D arrays showed an increase in localised red fluorescence intensity over 10 minutes, consistent with the formation of resorufin within individual coacervate microdroplets (Fig. 1c). Corresponding plots of the changes in intra-droplet green and red fluorescence intensity after sequential addition of heme and Amplex red/H_2_O_2_ confirmed that progressive activation of the microarray was correlated with heme binding to *apo*-mAPXmEGFP (Fig. 1d).

### Competitive heme-protein binding and modulated peroxidase activity

Having established that spatially distributed *apo*-mAPXmEGFP-containing coacervate microdroplets can be transformed by heme-activation into catalytically active microarrays, we sought to attenuate the concomitant *holo*-mAPXmEGFP enzyme activity by co-encapsulation within the molecular condensate droplets of a competitive heme-binding protein (Fig. 2a). For this, we used the *apo*-form of a double myoglobin variant (*apo*-gMb, H64Y/V68F,^32^*K*_d_ (heme) = 10^−11^ M; *apo*-mAPXmEGFP, *K*_d_ (heme) = 22 × 10^−9^ M),^11,33^ which has a much lower heme-bound peroxidase activity than *holo*-mAPXmEGFP (Fig. S9–S11 and Tables S1, S2).^34–36^ Control experiments in free solution confirmed that heme could be scavenged from *holo*-mAPXmEGFP by *apo*-gMb^37–39^ (Fig. S10). However, addition of *apo*-gMb to the acoustic chamber containing patterned coacervate microdroplets with sequestered pre-formed *holo*-mAPXmEGFP did not show heme scavenging due to molecular crowding of PDDA/ATP preventing direct protein–protein interactions and heme exchange (Fig. S12).

Based on these observations, we explored the feasibility of competitive heme-protein binding when encapsulated within the coacervate microdroplets. Injection of heme into a 2D array of coacervate droplets containing homogeneous distributions of Dylight-405-tagged *apo*-gMb and *apo*-mAPXmEGFP gave rise to minimal quenching of the *apo*-mAPXmEGFP green fluorescence, indicating preferential heme binding to co-encapsulated *apo*-gMb (Fig. 2b and c). A 1.5-times molar excess of *apo*-gMb was used to sequester the available heme, thereby preventing the formation of *holo*-mAPXmEGFP despite the initial excess of heme. Subsequent addition of H_2_O_2_ and Amplex red resulted in a negligible increase in resorufin concentration (Fig. 2c and S13), confirming that heme-activated biocatalytic activity within the condensate droplets was attenuated by competitive binding of the heme to *apo*-gMb. Although heme alone exhibited peroxidase activity, the rate and extent of output generation were approximately half that observed for *holo*-mAPXmEGFP, while remaining significantly higher and faster than that of *holo*-gMb (Fig. S6).

The above micro-array processor was subsequently integrated into a spatially distributed ternary population of coacervate droplets containing three different biocatalytic modules that could be chemically interconnected by inter-droplet diffusive signaling (Fig. 2d and e). The modules transformed a sucrose signal into a red fluorescent output in the presence of heme due to *holo*-mAPXmEGFP peroxidase activity (Fig. 2f). However, the output was minimal in the absence of heme (Fig. S6) and suppressed when *apo*-gMb was co-located with the *apo*-mAPXmEGFP module (Fig. 2f). Pre-programming the patterned coacervate droplets with *apo*-gMb inhibited output generation by competitively sequestering heme and thereby suppressing catalytic activity. The results indicated that long-range chemical signaling between the spatially isolated enzyme-containing coacervate droplet populations could be promoted or inhibited depending on specific heme-binding interactions with different proteins within the molecular condensates.

**Fig. 2:**
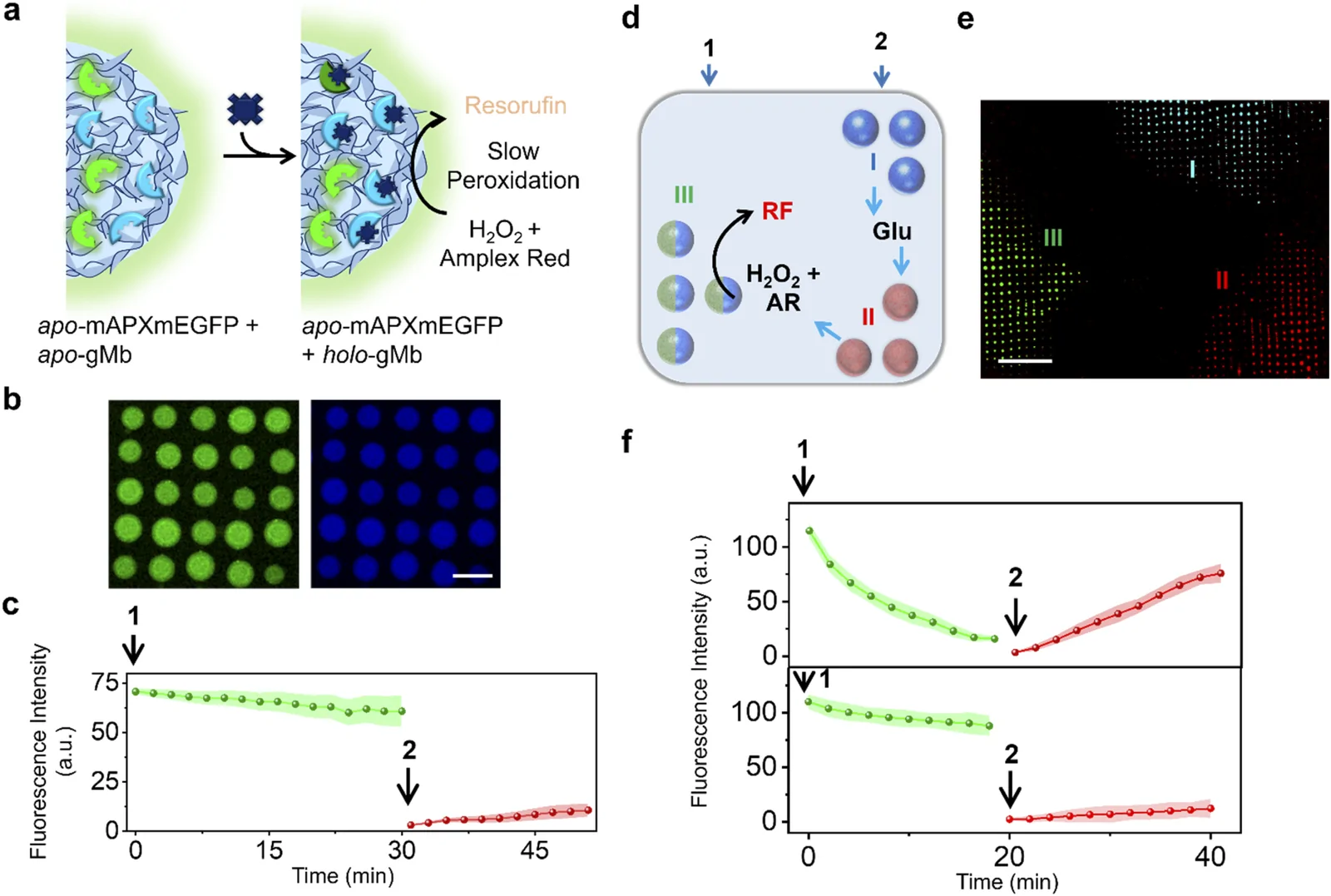
Competitive heme-protein binding and attenuated intra- and inter-droplet reactivity. (a) Scheme showing heme-induced selective protein activation in PDDA/ATP coacervate microdroplets containing *apo*-mAPXmEGFP (green) and *apo*-gMb (blue). Heme preferentially binds to *apo*-gMb to generate *holo*-gMb which exhibits slow peroxidase activity compared to *holo*-mAPXmEGFP. (b) Fluorescence microscopy images showing homogenous distribution of co-encapsulated *apo*-mAPXmEGFP (green; *λ*_ex_ = 450–490 nm; *λ*_em_ = 510 nm) and Dylight-405-tagged *apo*-gMb (blue; *λ*_ex_ = 355–425 nm; *λ*_em_ = 455) within 2D-patterned PDDA/ATP coacervate droplet microarrays. Scale bar, 150 µm. (c) Plot of time-dependent changes in fluorescence intensity recorded from coacervate droplets containing *apo*-mAPXmEGFP and *apo*-gMb after addition of heme (*t* = 0 min, arrow 1, green fluorescence) and H_2_O_2_/Amplex red (*t* = 31 min, arrow 2, red fluorescence). Fluorescence intensity measurements (a.u.) are collected from 5 × 5 grids of coacervate microdroplets; shaded error regions are obtained from triplicate experiments. (d and e) Schematic representation (d) and fluorescence microscopy image (e) of a spatially distributed ternary population of enzyme-containing PDDA/ATP coacervate droplets containing three different biocatalytic modules. Module I, sucrose input processor/glucose transmitter, Dylight 405-invertase (blue fluorescence); Module II, glucose processor/hydrogen peroxide transmitter, RITC-GOx (red fluorescence 2); Module III, peroxide processor/output generator, *apo*-mAPXmEGFP (green fluorescence) or co-encapsulated *apo*-mAPXmEGFP and *apo*-gMb. Heme (arrow 1), invertase (Inv), sucrose (Suc, arrow 2), glucose (Glu), Amplex red (AR), resorufin fluorescence (RF). Scale bar, 500 µm. (f) Plot of time-dependent changes in fluorescence intensity recorded from coacervate droplets of Module III without (top profile) or with *apo*-gMb (bottom profile) after addition of heme (*t* = 0 min, arrow 1, green fluorescence) and sucrose/Amplex red (*t* = 21 min, arrow 2, red fluorescence). Communication between the three modules gives a significant red fluorescence readout (resorufin) in the absence of *apo*-gMb in Module III. Fluorescence intensity measurements (a.u.) are collected from 5 × 5 grids of coacervate microdroplets; shaded error regions are obtained from triplicate experiments.

### Heme-triggered chemical communication across synthetic-living coacervate networks

Inspired by the above observations, we sought to use heme-sensitive living cells as efficient, self-contained catalytic systems for regulating biocatalytic reactions in molecular condensates. Specifically, we investigated heme-mediated chemical signaling within PDDA/ATP coacervate microdroplets containing *E. coli* bacterial cells capable of expressing the *apo*-mAPXmEGFP fusion protein (*apo*-mAPXmEGFP-*E. coli*) (Fig. 3a). Confocal fluorescence microscopy images showed that the bacterial cells were readily sequestered within individual coacervate droplets (Fig. 3b) with typical loadings of *ca*. 40 cells per microdroplet (Fig. S14). Cell viability studies showed that over 80% of the sequestered bacterial cells were alive for up to five hours and that the analytes had negligible effect on the viability (Fig. S15). Significantly, green fluorescence associated with the *apo*-mAPXmEGFP-*E. coli* cells was quenched over a period of 40 min when aqueous heme was added to a suspension (Fig. S15) or a patterned 2D microarray (Fig. 3c and S16) of bacteria-containing molecular condensates. The results were consistent with heme diffusion across the bacterial cell wall^40–42^ and subsequent binding to intracellular *apo*-mAPXmEGFP, resulting in the formation of *holo*-mAPXmEGFP within the bacterial cells. Subsequent addition of H_2_O_2_ and Amplex red gave rise to resorufin red fluorescence within the microdroplet arrays, confirming that the intracellular *holo*-mAPXmEGFP was enzymatically active (Fig. S17).

**Fig. 3:**
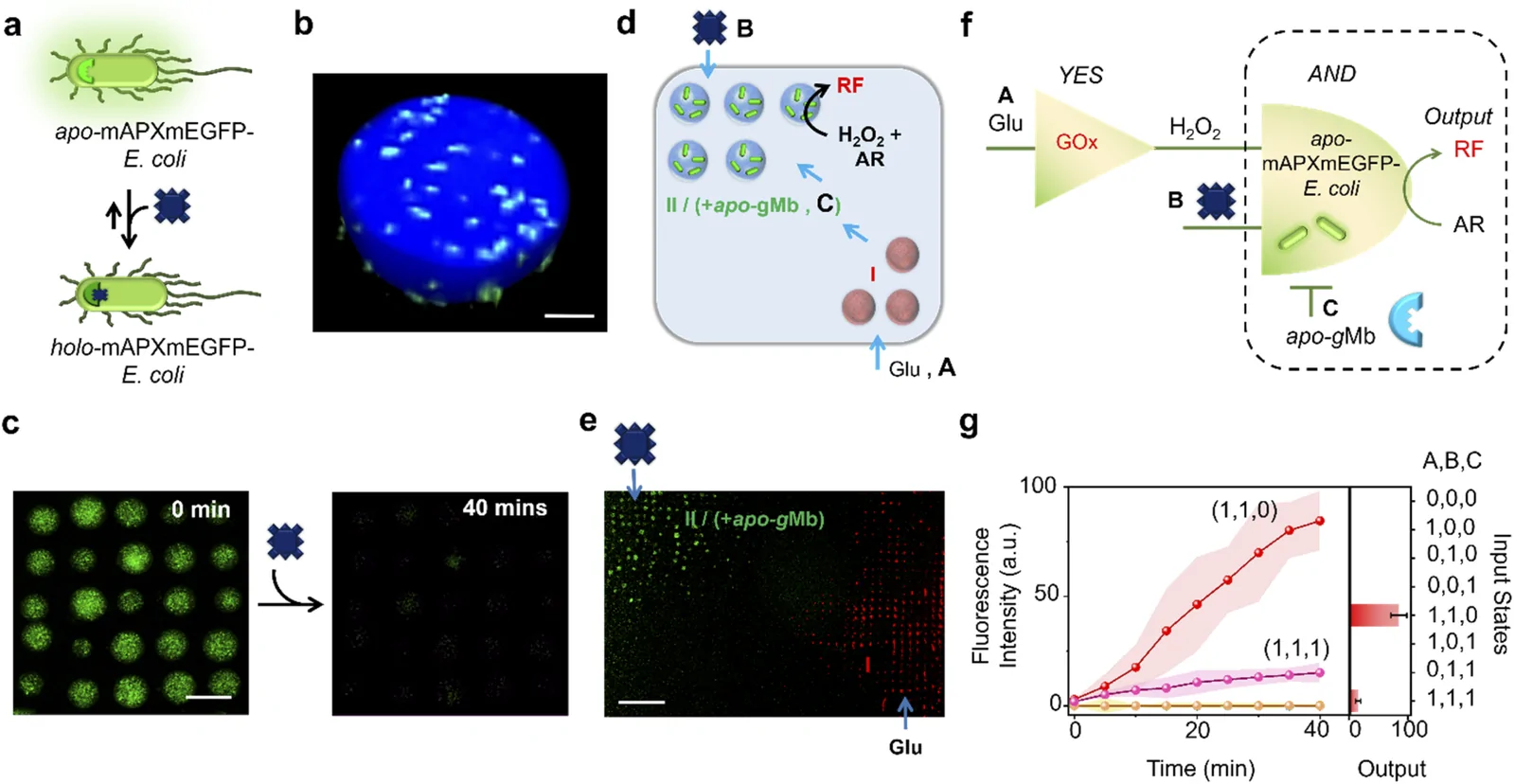
Heme-mediated chemical signaling in synthetic/living condensate droplet networks. (a) Schematic representation of heme-induced fluorescence quenching in *apo*-mAPXmEGFP-*E. coli* cells. (b) Z-stacked confocal fluorescence microscopy image of PDDA/ATP coacervate droplets loaded with Dylight-405-*apo*-gMb (blue fluorescence) and *apo*-mAPXmEGFP-*E. coli* cells (green fluorescence). Scale bar, 50 µm. (c) Fluorescence microscopy images before (*t* = 0 min) and 40 min after addition of heme to an acoustically patterned array of coacervate droplets containing *apo*-mAPXmEGFP-*E. coli* cells showing fluorescence quenching due to heme uptake and binding to intracellular *apo*-mAPXmEGFP, resulting in the formation of *holo*-mAPXmEGFP within the bacterial cells. Scale bar, 100 µm. (d and e) Schematic representation (d) and fluorescence microscopy image (e) of a spatially distributed binary population of PDDA/ATP coacervate droplets containing two different biocatalytic modules. Module I, RITC-GOx (red fluorescence, YES gate, signal generator); Module II, *apo*-mAPXmEGFP-*E. coli* cells (green fluorescence) without or with *apo*-gMb (C) (AND gate, signal effector). Glucose (A, Glu), heme (B, blue symbol), Amplex red (AR), resorufin fluorescence (RF), hydrogen peroxide (H_2_O_2_). Scale bar, 500 µm. (f) Schematic representation of a droplet-embodied concatenated biohybrid YES → AND through-space logic circuit. An RF output is observed only if heme and *apo*-gMb are present and absent, respectively. (g) Plots of time-dependent changes in fluorescence output intensity recorded from coacervate droplets of Module II with different input combinations and corresponding final output bar charts obtained for the through-space YES → AND circuit described in (f). Glucose (A), heme (B) and *apo*-gMb (C) are used as inputs (A–C). Output state 1 is observed only for the input state (1,1,0) red curve. For input state 1,1,1 (magenta curve) the output observed is >0 due to incomplete inhibition. The fluorescence-intensity outputs (a.u.) were processed from a 5 × 5 grid of coacervate microdroplets. Shaded error regions were obtained from triplicate experiments, and graphical data represent the mean ± SD.

We used the above system and the attendant capacity for implementing heme signaling and scavenging to construct a bacterial cell/protocell-mediated biochemical circuit capable of spatially distributed logic gate operations.^43^ Specifically, we produced a through-space concatenated YES → AND embodied logic circuit by constructing 2D micropatterned droplet arrays consisting of two spatially separated populations of PDDA/ATP coacervate microdroplets capable of diffusive chemical communication and production of a red fluorescent output only if heme and *apo*-gMb were present and absent, respectively (Fig. 3d and e). In contrast, the fluorescence output was attenuated when all three inputs (glucose, heme and *apo*-gMb) were added due to competitive binding of heme to *apo*-gMb and switching off intracellular *holo*-mAPXmEGFP activity in the *E. coli* cells (Fig. 3f, g and S17). All other input combinations gave no fluorescent read-out as the absence of either glucose or heme, or both, failed to execute the concatenated YES → AND logic operation, confirming that the circuit only operated when a heme-bound protein efficiently consumes enzymatically generated H_2_O_2_ within the molecular condensates.

## Conclusions

This study presents a step towards understanding how competitive protein interactions regulate heme binding, exchange and distribution within molecularly crowded cytomimetic environments. By tuning these interactions, it is possible to achieve spatial and temporal control over heme-dependent enzyme activities by diffusive signaling between populations of patterned coacervate droplets. This approach is extended to biohybrid systems comprising segregated populations of living and synthetic cells capable together of emulating cellular functions and performing logic-based operations in response to defined input signals and heme-mediated signaling/activation. In this context, our studies contribute to the broader vision of constructing programmable, self-regulating biomimetic environments for future protocell-based therapeutic, diagnostic and computational technologies.

In a wider context, we propose that condensate droplets are suitable molecularly crowded liquid phase-separated platforms for the reconstitution and modelling of intracellular heme-mediated processes. The capacity to sequester heme indicates that coacervate droplets are useful media for investigating protein-mediated heme binding and regulation, offering insights into how the distribution and availability of intracellular heme could potentially be managed in cells responsive to environmental changes. Competitive heme-binding proteins enable programmable control of enzyme activity through dynamic cofactor availability. Specifically, we propose that the uptake and enrichment of heme in coacervate microdroplets could provide a useful model for investigating alternative heme homeostatic mechanisms. We further speculate that reversible sequestration and dynamic exchange between intracellular biomolecular condensates may contribute to the spatial and temporal regulation of heme availability in biological systems. In this view, introducing donor and acceptor proteins and/or heme chaperones that can dynamically regulate the transfer of labile heme or free cellular heme pools would enable a controlled heme-sequestration system, in which heme can be selectively captured, retained and subsequently released on demand. Such a responsive protein-based mechanism would significantly enhance the functionality and versatility of the coacervate-based microarrays. Extending this strategy to other cofactor-regulating proteins and hybrid artificial-living cell systems could provide a versatile platform for biochemical computing in engineered cells.

## Experimental

### Materials

The following materials were supplied by Sigma Aldrich and used as received: rhodamine B isothiocyanate (RITC), fluorescein isothiocyanate (FITC), glucose oxidase from aspergillus niger (GOx, Type XS, 150 k units per g), invertase from baker's yeast *S. cerevisiae* (Inv, 200–300 units per mg solid), poly(diallyldimethylammonium chloride) (PDDA, 20 wt%, *M*_w_ ≈ 100–200 kDa), adenosine 5-triphosphate (ATP), hydrogen peroxide (H_2_O_2_) 30% w/v, glucose, sucrose, heme, propidium iodide, Dylight 405 and Amplex red were purchased from Thermo Fisher Scientific and dimethyl sulfoxide (DMSO) was purchased from Fisher Chemical.

#### Heme binding experiments with *apo*-mAPXmEGFP and *apo*-gMb

Buffer-exchanged samples of *apo*-mAPXmEGFP and *apo*-gMb were mixed with 25% v/v and 10% v/v glycerol, respectively, as a cryoprotectant for storage at −80 °C. The stock concentrations of *apo*-mAPXmEGFP and *apo*-gMb were maintained at 47 µM and 14 µM, respectively, with further dilutions made for binding experiments in which known concentrations of *apo*-mAPXmEGFP or *apo*-gMb in water were titrated against heme and monitored by UV-vis and fluorescence measurements.

#### Partition coefficients of proteins (*apo*-mAPXmEGFP and *apo*-gMb) and heme

*apo*-mAPXmEGFP and Dylight-405 *apo*-gMb-containing patterned PDDA/ATP coacervates were prepared following the standard procedure. To determine the partition coefficient (*K*), the excess supernatant was removed. Fluorescence microscopy images of the protein-containing coacervate microdroplets were recorded and the fluorescence intensities measured by ImageJ software. The partition coefficient was experimentally determined as the ratio between the fluorescence intensity of the coacervate and the fluorescence intensity of the supernatant.^44^ At total concentrations of 5 µM *apo*-mAPXmEGFP and 14 µM *apo*-gMb, the partition coefficients in the PDDA/ATP coacervate were 2.72 and 3.21, respectively. The partition coefficient of heme in the PDDA/ATP coacervates was determined from UV-vis spectra by monitoring the absorbance at *λ*_385nm_. At a total heme concentration of 30 µM and pH 8, the partition constant was 5.62 (Table S3).

#### Heme binding and peroxidase reactions of *apo*-mAPXmEGFP or *apo*-gMb in coacervate droplets

A 5 µM solution of *apo*-mAPXmEGFP or *apo*-gMb was added to a solution of PDDA (50 mM, 250 µL, pH 8), followed by the addition of ATP (50 mM, 250 µL, pH 8). The resulting *apo*-mAPXmEGFP-loaded PDDA/ATP coacervates were centrifuged to remove excess unencapsulated protein, and the supernatant was replaced with water. The coacervate phase was then redispersed in water (pH 8) and deposited onto a coverslip. A 30 µM solution of heme was introduced into the coacervate solution containing encapsulated *apo*-mAPXmEGFP, *apo*-gMb, or both, and time-dependent fluorescence changes were monitored using a fluorescence microscope. Peroxidase reactions were triggered by introducing Amplex red (10 mM) and H_2_O_2_ (25 mM) and the fluorescence increase as a result of resorufin formation was monitored over time.

#### Acoustic patterning^45^ of *apo*-mAPXmEGFP or *apo*-gMb encapsulated PDDA/ATP coacervates

For the formation of 2D arrays of PDDA/ATP coacervate microdroplets, orthogonal transducer pairs were operated at slightly different frequencies during the trapping process. This setup created a grid of acoustic potential minima, causing relatively dense objects to migrate towards these points due to acoustic radiation forces. The transducer pairs were run near the third harmonic resonance frequency of the piezoelectric plates (6.76/6.78 MHz, 10 V). Patterned PDDA/ATP coacervate microdroplets were generated *in situ* by adding an aqueous ATP solution (150 µL, 50 mM, pH 8) to an aqueous PDDA (300 µL, 25 mM monomer, 100–200 kDa, pH 8) and protein (5 µM of *apo*-mAPXmEGFP, 14 µM *apo*-gMb or both) solution. The mixture was left undisturbed for 20 min to allow homogeneous coacervate droplet formation through continuous coalescence in the square chamber, resulting in 2D microdroplet array. After completion of coacervate formation, the supernatant was exchanged with water (pH 8) twice.

#### Heme induced activation and peroxidase reactions in acoustically trapped droplets with *apo*-mAPXmEGFP or *apo*-gMb

Acoustically patterned PDDA/ATP coacervates loaded with *apo*-mAPXmEGFP (5 µM) or *apo*-gMb (14 µM) were prepared following which heme was introduced keeping the final concentration as 30 µM. The fluorescence changes were monitored over time. Amplex red (10 mM, 10 µL) and H_2_O_2_ (25 mM, 10 µL) was later added into the sample chamber and the time dependent red fluorescence evolution corresponding to resorufin was monitored through fluorescence microscopy.

#### Construction of acoustically patterned protocell modules and logical response from inter-modular communication

The construction of protocell modules in the acoustic chamber was carried out following the previously reported technique.^29^ The multicomponent arrays of PDDA/ATP coacervate droplets were prepared with communicating populations of proteins such that they are well spaced within the observation window. After switching off the acoustic field, the inputs are introduced carefully, 5–6 mm from the sample viewing area close to the sender module to prevent any disturbance to the patterned modules. Heme is introduced to the effector module loaded with *apo*-mAPXmEGFP or *apo*-mAPXmEGFP and *apo*-gMb and incubated for 20 min. The reaction products diffuse from the sender module to transmitter and finally to the effector module. Fluorescence outputs are collected over time by zooming into the final effector module.

#### Entrapment of *apo*-mAPXmEGFP-*E. coli* inside PDDA/ATP coacervates

*E. coli* cells expressing *apo*-mAPXmEGFP (*apo*-mAPXmEGFP-*E. coli*) were cultured overnight until they reached the saturation phase, achieving an OD_600_ of 1.82. The bacterial cells were then harvested by centrifugation at 10 000 rpm for 10 minutes and resuspended in LB broth to a final concentration of OD_600_ = 20. To initiate coacervate formation, 3 µL of the *apo*-mAPXmEGFP-*E. coli* suspension was added to 50 µL of PDDA (50 mM), followed by 50 µL of ATP (50 mM). The mixture was gently shaken by hand for 1 minute, resulting in the formation of coacervates encapsulating the *E. coli* cells. The aqueous dispersion of PDDA/ATP coacervates was then centrifuged at 10 000 rpm for 2 minutes to separate the coacervate phase. After removing the supernatant, 60 µL of water and 40 µL of LB broth were added, and the *E. coli*-containing coacervates were gently redispersed using a pipette. The resulting suspension was incubated for 5 minutes to ensure complete engulfment of the bacterial cells.

#### Heme induced programmable activation of *apo*-mAPXmEGFP-*E. coli*-loaded patterned coacervates

50 µL aliquot of preformed *apo*-mAPXmEGFP-*E. coli*-loaded PDDA/ATP coacervates was introduced into the acoustic chamber, where transducer pairs operating at 6.76/6.78 MHz and 10 V induced spatial patterning of the coacervates. Subsequently, heme (30 µM) was added and incubated for 40 minutes, during which fluorescence changes were monitored over time causing heme-induced quenching of *apo*-mAPXmEGFP-*E. coli* within the coacervates. *apo*-gMb was co-encapsulated with *apo*-mAPXmEGFP-*E. coli* into PDDA/ATP coacervates to enable programmable protein activation. Peroxidase activity was initiated by introducing Amplex red (10 mM, 10 µL) and H_2_O_2_ (25 mM, 10 µL). Fluorescence microscopy images were captured at the centre of the trapping chamber within the designated observation window.

#### Construction of acoustically patterned *apo*-mAPXmEGFP-*E*. *coli*-loaded protocell modules and logical response from inter-modular communication

*apo*-mAPXmEGFP-*E. coli* or a combination of *apo*-mAPXmEGFP-*E. coli* and *apo*-gMb-loaded PDDA/ATP coacervate modules were prepared by introducing 4 µL of the preassembled solution into the sample chamber, thereby establishing a localized population of coacervate containing bacterial cells. These modules serve as output-forming effectors, with heme acting as the input signal. The sender module was prepared separately by introducing 4 µL of a GOx + PDDA mixture into an ATP-loaded acoustic chamber. Upon addition of glucose as input to the sender module, hydrogen peroxide is generated and functions as a messenger molecule. Fluorescence outputs were monitored over time by focusing on the final effector module.

## Author contributions

A. J. P, E. L. R. and S. M. conceived and supervised the research project. A. M., A. E. G. and A. J. P. discussed and designed the experiments. A. M. and A. E. G. performed the experiments and data analysis. All authors discussed the data and contributed to the manuscript preparation.

## Conflicts of interest

There are no conflicts to declare.

## Supplementary Material

SC-OLF-D6SC06692E-s001

## Data Availability

The data supporting this article have been included as part of the supplementary information (SI). Any additional information that support the findings of this study are available from the corresponding author upon reasonable request. Supplementary information is available. See DOI: https://doi.org/10.1039/d6sc06692e.
