# Supplementary material for: Heme sequestration and signaling in molecular condensates
Source: Chem Sci. 2026 Oct 22. Online ahead of print. doi: 10.1039/d6sc06692e (PMC13688254; doi:10.1039/d6sc06692e)
Supplement: Supplementary file 1 [file SC-OLF-D6SC06692E-s001.pdf]

Supplementary Information (SI) for Chemical Science.
This journal is © The Royal Society of Chemistry

Heme sequestration and signaling in molecular condensates

Ananya Mishra†,[a,c] Andrea E. Gallio†,[b] Emma L. Raven*,[b] Avinash J. Patil*[a] and Stephen Mann*[a,c]

a Centre for Protolife Research and Centre for Organized Matter Chemistry, School of Chemistry, University of Bristol, Bristol, BS8 1YS, United Kingdom

b School of Chemistry, University of Bristol, Bristol, BS8 1TS, United Kingdom

c Max Planck-Bristol Centre for Minimal Biology, School of Chemistry, University of Bristol, Bristol, BS8 1TS, United Kingdom

† Authors contributed equally to the work

1. Supplementary experimental section

Preparation of heme stock solution

Heme solutions were prepared in water by adding 50 μL of 0.1 M NaOH to a 1.5 mL vial containing a micro-spatula tip loaded with heme. The solution was shaken for 30 sec, followed by dilution with 1 mL of H_2O to produce a suspension. The suspension was centrifuged at 12000 rpm for 1-2 min and the heme-containing supernatant removed into another vial and further diluted. The above steps were repeated twice until no heme sediment was observed in the centrifugation step. The concentration of heme solution was calculated by UV-Vis spectra ($\square_{heme}$ (385 nm) = 58400 M^{-1} cm^{-1}).

Components for the expression and purification of mAPXmEGFP and gMb (sperm whale myoglobin H64Y/V68F) from E. coli BL21(DH3)

The following materials were supplied by the specified manufacturers and used without further purification: ampicillin (Sigma), Lysogeny Broth powder (Melford), isopropyl-D-1- thiogalactopyranoside (IPTG; ChemCruz), KH_2PO_4 (Melford), KCl (Sigma), deoxyribonuclease I (DNase I from bovine pancreas; Roche), EDTA-free protease inhibitor cocktail (Roche), lysozyme (Sigma), succinylacetone (SA; Sigma Aldrich), Fe-protoporphyrin IX (Fluka), ammonium sulfate (Fischer Bioreagent), DEAE-Sepharose (Sigma), InstantBlueTM Coomassie stain (Expedeon), dithiothreitol (DTT; Thermo Scientific).

Expression and purification of fusion protein mAPXmEGFP

The mAPXmEGFP expression construct encoding monomeric APX fused to monomeric EGFP was contained in a pLEICS45 vector and generated by PROTEX (University of Leicester).[1] The recombinant expression of *apo*-mAPXmEGFP was carried out in LB medium supplemented with 1 mM succinylacetone (an inhibitor of heme biosynthesis, as previously described).[2] Briefly, expression in *E. coli* BL21(DE3) was induced in 2L baffled Erlenmeyer flasks with 0.5 mM isopropyl -D-1- thiogalactopyranoside (IPTG) and by dropping the temperature to 23 °C when OD_{600} was 0.4-0.7. Cells were harvested 20 h post-induction, lysed, and purified by Immobilised Metal Affinity Chromatography (IMAC; HisTrap excel 5 mL), buffer exchange

and, finally, size exclusion chromatography using a HiLoad Superdex 16/600 200 pg column) and 10 mM K_2HPO_4, 150 mM KCl, pH 7 elution buffer. Purified mAPXmEGFP samples were fractionated and stored at -80°C in elution buffer containing 25% *v*/*v* glycerol.

Expression and purification of sperm whale myoglobin H64Y/V68F (gMb)

The pEMBL19 vector (pEMBL19-gMb) for the recombinant expression of gMb (sperm whale myoglobin H64Y/V68F) was originally developed by Olsen and co-workers.[1] The expression protocol was adapted from established methods previously reported in the literature.[2,3] Briefly, *E. coli* BL21(DE3) cells expressing gMb were harvested 20 h post-induction and lysed. The homogenate was first enriched in gMb by salting out purification, in a two-stages. Ammonium sulphate was slowly added 40% m/V and the suspension incubated at 4 °C for 2 h. The suspension was centrifuged (4000 rpm, 10 min) and further ammonium sulphate was slowly added to the supernatant to 20% m/V (concentration zeroed to the previous addition). The suspension was incubated at 4 °C for 2 h and centrifuged (4000 rpm, 10 min). The supernatant was dialysed twice (20 mM Tris, 1 mM EDTA, pH 8) and then purified by size-exclusion chromatography using a HiLoad 16/600 Superdex 75 column (elution buffer: 20 mM Tris, 1 mM EDTA, pH 9). Fractions containing gMb were identified by SDS-PAGE, pooled, concentrated, and applied to a DEAE-Sepharose ion exchange column equilibrated in 20 mM Tris, 1 mM EDTA, pH 8.4. Purified gMb samples were fractionated and stored at -80°C in 10% *v*/*v* glycerol.

Extraction of heme from gMb to produce *apo*-gMb

The procedure was adapted from Teale's method.[4] In a glass test tube, an ice-cold 1 mL aliquot of ∼100 μM Mb was acidified by addition of 0.1 M HCl drop-by-drop until the dark green solution turned bright pink. Approximately 1 mL of ice-cold butanone was added. The organic and aqueous phases were shaken, and the test tube was incubated at 0 °C for 10 min. A pink butanone layer was obtained over a colourless aqueous layer. The aqueous layer containing *apo*-gMb was isolated and dialysed and dialysed to remove to remove traces of ketone. Multiple dialysis steps were performed against

1 – 6 mM $NaHCO_3$, 1 mM EDTA (12h, 4°C).

2 – 6 mM $NaHCO_3$ (12h, 4°C). Repeated twice against fresh buffer following the first round.

3 – 50 mM K_2HPO_4, pH 7 (4°C).

Successful heme extraction was confirmed by UV-Vis.

Spectroscopy

UV/Vis spectroscopy measurements were carried out in a double-beam Perkin Elmer Lambda40, Perkin Elmer Lambda750 and the fluorescence experiments were carried out using a CLARIO star Plus fluorescence microplate reader.

Calculation of catalytic efficiency of proteins

In a 1 mL quartz cuvette, 33 μM L-ascorbic acid and 116 nM APx were pre-incubated (25 °C) for 3 minutes in 10 mM K_2PO_4, 150 KCl, pH 7. The reaction was initiated by the addition of H_2O_2 (2.5 μL, 30 mM). Oxidation of ascorbate into monodehydroascorbate (MDA) was monitored by obtaining time-dependent

spectra of the decay in the absorbance at 290 nm. The reaction catalysed is: 2 ascorbate + $H_2O_2 \rightarrow$ 2 MDA + 2 H_2O, where the catalyst is *apo*-mAPXmEGFP, *holo*-mAPXmEGFP, gMb, or heme. Enzyme concentrations were calculated from dilution of a stock of known absorbance concentration. Peroxidase activities measured under the assay condition were calculated as specific activities (s^{-1}) normalised to catalyst concentration from the slope of the initial decay in the absorbance at 290 nm, according to the equation:

$$\text{Peroxidase activity} = \text{slope} \times \frac{1}{\varepsilon_{\text{ascorbate (290 nm)}}} \times \frac{1}{[\text{catalyst}]}$$

Slopes were obtained from the time-dependent data using a linear least square fit from the initial reaction rate to estimate catalytic activity. For L-ascorbic acid, ε_{290} = 2.8 $mM^{-1}cm^{-1}$ was used throughout. The reported peroxidase activities are shown in Table S1.

Fluorescence and confocal fluorescence microscopy

Fluorescence imaging was conducted using a Leica DFC 310FX, and Confocal Scanning Laser Microscopy (CSLM) was performed with a Leica SP5-II confocal laser scanning microscope, connected to a Leica DMI 6000 inverted epifluorescence microscope. The setup included a 50-mW laser (405 nm), a 150-mW Ar laser (488 nm), and a 20-mW solid-state yellow laser (561 nm), along with 20X, 40X, or 63X objectives. Dye molecules were excited using specific filters with designated excitation (λ_{ex}) and emission (λ_{em}) wavelength cutoffs. 3D images were acquired through z-stacks with 0.1 μm slices. All captured images were analysed and processed using FIJI ImageJ software. For fluorescence imaging: Dylight 405 λ_{ex} = 355 - 425 nm and λ_{em} = 455 nm, *apo*-mAPXmEGFP, λ_{ex} = 450 - 490 nm and λ_{em} = 510 nm; RITC, Propidium Iodide and Amplex red, λ_{ex} = 515 - 560 nm and λ_{em} = 580 nm. For CLSM: Dylight405 λ_{ex} = 405 λ_{em}= 415-460 nm, RITC and Propidium Iodide λ_{ex}= 561 λ_{em}= 571-630 nm, *apo*-mAPXmEGFP λ_{ex}= 488 λ_{em}= 498-550 nm.

Pegylation of coverslips

The coverslips were pre-cleaned by sonication in 70% ethanol for 5 minutes, followed by immediate drying with nitrogen, and placed in a staining jar. A solution of 10 mL toluene and 200 μL PEG-silane was added to the jar under an argon atmosphere. After 24 hours of incubation, the slides were individually rinsed with ethanol and air-dried.

Acoustic device

A custom-built acoustic trapping device based on a square arrangement of four piezoelectric transducers (Noliac, NCE 51, L15 x W2mm x T1 mm) was used. The opposing transducer pairs were wired in series, driven by two signal generators (Agilent 33220a-001), and connected to an oscilloscope (Agilent DSOX2014A). A PEG-functionalised glass coverslip was attached to the bottom of the device using an adhesive.

Fluorescent labelling of proteins

The enzymes (20 mg) were dissolved in sodium carbonate buffered solutions (7 mL, 0.1 M, pH 8.5). Dye solution of RITC dissolved in dimethyl sulfoxide (DMSO) and Dylight 405 in water (200 μL, 1 mg mL^{-1}) was added dropwise into the enzyme solution. The reaction mixtures were kept at 4 °C for 12 h and then dialysed (molecular weight cutoff 15 kDa) against Milli-Q water over two days with regular water changes. The tagged enzymes were then lyophilized and stored in the dark before use.

Scavenging of heme from *holo*-mAPXmEGFP APx by *apo*-gMb in solution

For the heme exchange assay between *holo*-mAPXmEGFP and *apo*-gMb, varying amounts of *apo*-gMb were added to a constant concentration of *holo*-mAPXmEGFP. The assay was conducted in a clear, flat-bottom 96-well plate (Greiner), with 1 μM *holo*-mAPXmEGFP incubated with increasing molar ratios of *apo*-gMb in separate wells, in a total reaction volume of 100 μL per well (Buffer: 50 mM KH_2PO_4, 100 mM KCl, pH 7.4). The [*apo*-gMb]/[*holo*-mAPXmEGFP] molar ratios ranged from 0 to 10, increasing in 0.5 equivalents. The plate was incubated for 20 minutes at room temperature, and the fluorescence intensity of *holo*-mAPXmEGFP was measured with a CLARIOstar Plus fluorescence microplate reader ($λ_{ex}$ = 480-10 nm; $λ_{em}$ = 504-554 nm).

Viability experiments of *apo*-mAPXmEGFP-*E. coli* in PDDA/ATP coacervates

Aliquots of PDDA/ATP coacervates solution loaded with *apo*-mAPXmEGFP-*E. coli* (50 μL) were removed at different time points and 1 μL of propidium iodide (20 mM stock in DMSO) was introduced to it. The mixture was kept at 0 °C for 15 mins before analysing the samples under confocal fluorescence microscopy. All the *apo*-mAPXmEGFP-*E. coli* cells exhibited green fluorescence while only dead cells showed red fluorescence.

Calibration of the OD_{600} spectrophotometer and calculation of *E. coli* cells trapped per protocell

A suspension culture of the *E. coli* BL-21(DE3) expressing *apo*-mAPXmEGFP-*E. coli* was prepared as outlined above. After 20 h post-induction with IPTG, the culture was diluted to obtain a series of samples with optical densities (ODs) of 1.82, 1.32, 1.02, 0.67, 0.48, 0.35, and 0.2, respectively. OD_{600} values were measured using a Biochrom Ultrospec 10 cell density meter. For each OD value, dilutions of 1 in 10^6 were prepared for plating onto LB-agar plates containing 100 μg/mL ampicillin and incubated overnight at 37 °C. Following overnight incubation, the colonies formed in each plate were counted to determine the Colony Forming Unit(s) (CFU). CFUs were then multiplied by the dilution factor and plotted against the respective OD_{600} values to build a calibration curve. The gradient of the linear portion of the curve was used to determine the conversion factor OD_{600} (1) = 3 x 10^7 cells/mL. The concentration of *E.coli* cells is diluted to 6 x 10^3 cells/μL in the coacervate solution. When 4 μL of coacervate solution is deposited carefully into the field of view of acoustic chamber, it results in the formation of □600 patterned coacervates. This gives □40 *apo*-mAPXmEGFP-*E. coli* cells/coacervate droplet and □32 live *apo*-mAPXmEGFP-*E. coli* cells/coacervate droplet.

2. Supplementary Figures

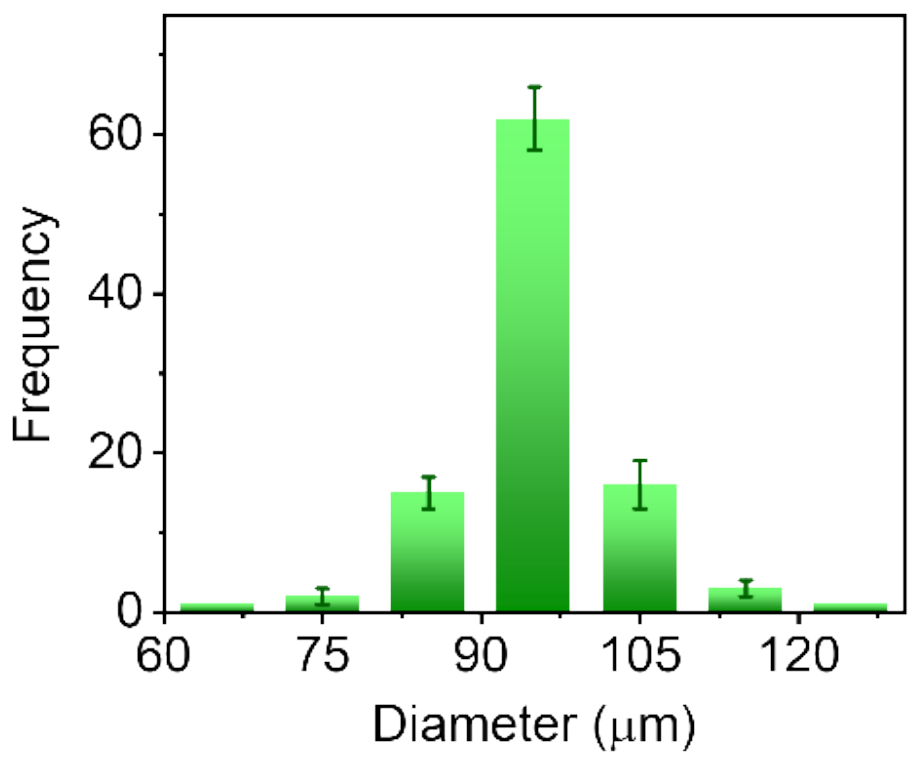

Supplementary Fig. S1: Size distribution of acoustically patterned coacervates. Size distribution of acoustically patterned coacervates. Size distribution of coacervates obtained from three independent sets of 10 × 10 coacervates (100 droplets per set). More than 60% of the coacervates had diameters between 90 and 100 μm. A small fraction of coacervates exhibited slight deviations in size, likely due to incomplete or non-uniform coalescence during the patterning.

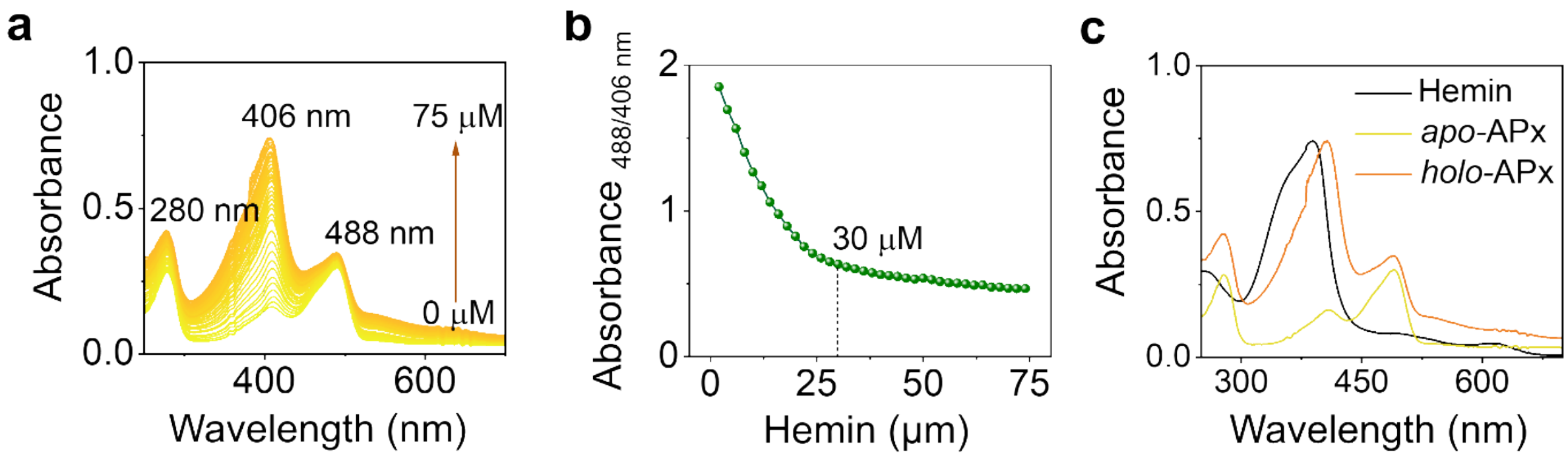

Supplementary Fig. S2: Spectroscopic data showing heme binding to *apo*-APX. a) Absorption spectra and b) titration curve of *apo*-mAPXmEGFP solution (5 μM) with increasing concentrations of heme (0 μM to 75 μM). c) Comparative absorption spectra of 5 μM of *apo*-mAPXmEGFP, 5 μM of *holo*-mAPXmEGFP and 30 μM of heme. The spectroscopic data confirms the enhancement in the Soret band intensity (Absorbance$_{488/406\ nm}$) with each successive addition of heme which saturation at 30 μM.

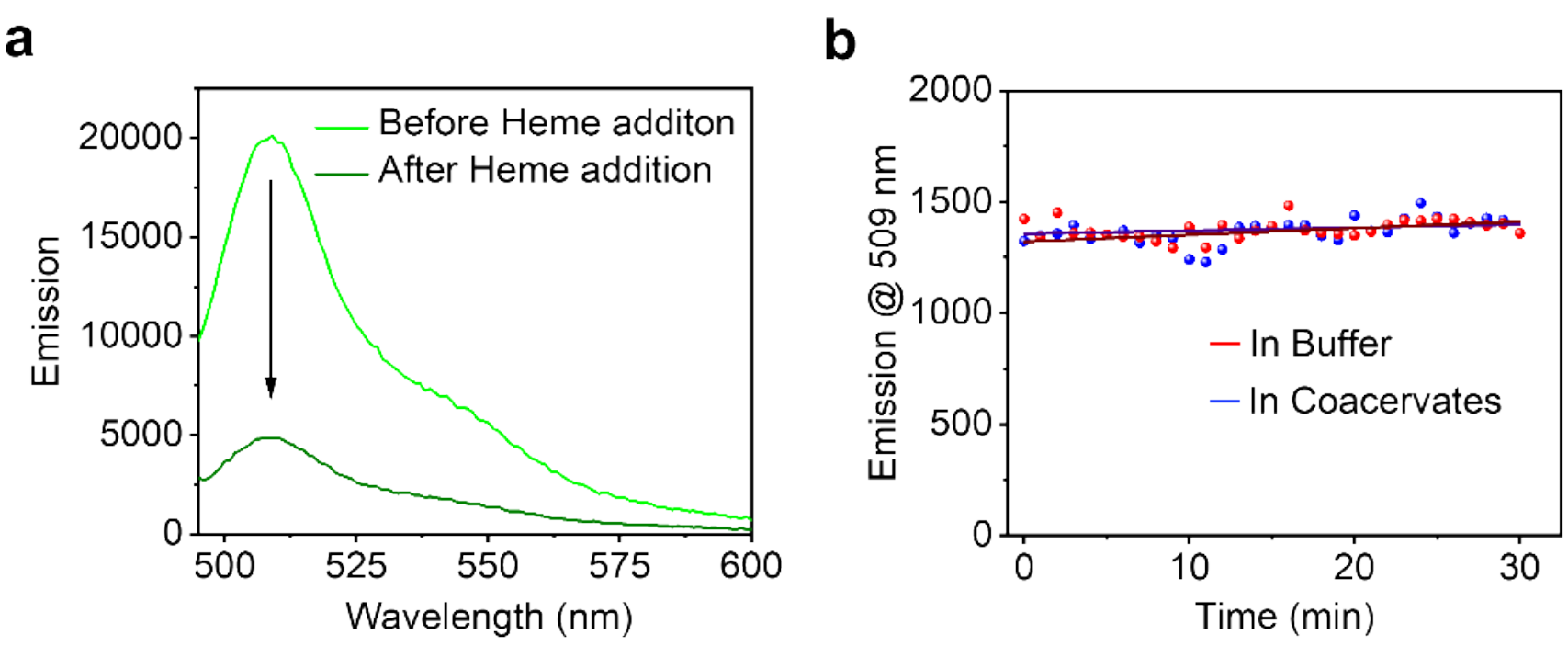

Supplementary Fig. S3: Heme-mediated fluorescence quenching of *apo*-mAPXmEGFP a) Change in emission intensity of coacervate droplet-sequestered *apo*-mAPXmEGFP (14 μM) upon addition of heme (30

μM). b) Time-dependent emission changes over 30 mins when heme is added to bulk *apo*-mAPXmEGFP solution (red circles) or to coacervate microdroplets containing *apo*-mAPXmEGFPs (blue circles). $□_{ex}$ = 488 nm, $□_{em}$ = 510 nm).

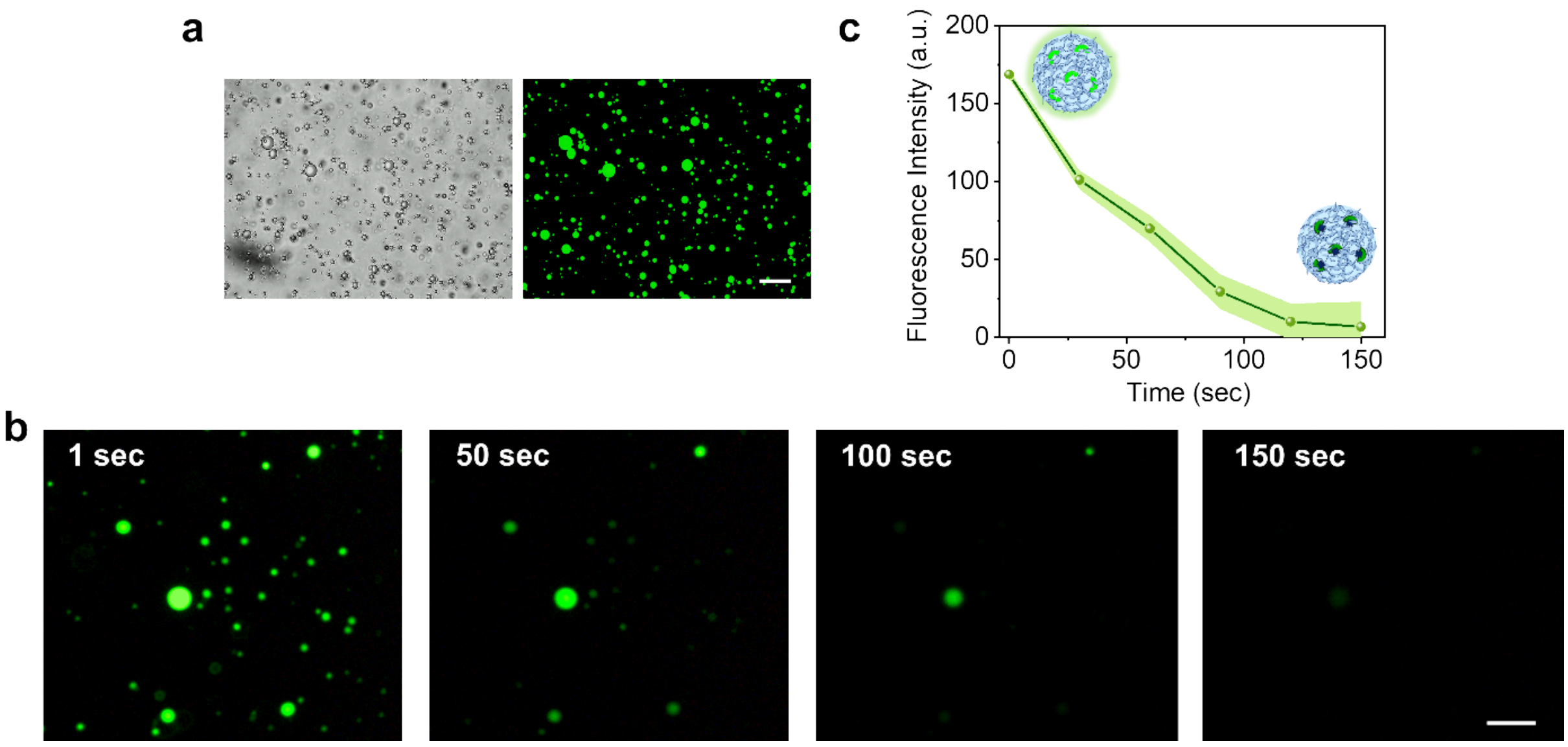

Supplementary Fig. S4: Sequestration and heme-mediated fluorescence quenching of *apo*-mAPXmEGFP in free suspensions of molecular condensates. a) Brightfield and fluorescence microscopy images of PDDA/ATP coacervates loaded with *apo*-mAPXmEGFP ($λ_{ex}$ = 450-490 nm and $λ_{em\ cutoff}$ = 510 nm, Scale bar- 100 μm). The samples were prepared in the absence of an acoustic pressure field. b) Corresponding time-lapse fluorescent microscopy images (Scale bar- 20 μm) and c) fluorescence changes over time showing fluorescence quenching of free PDDA/ATP coacervates loaded with *apo*-mAPXmEGFP (5 μM) on addition of 30 μM heme. Inset shows scheme of fluorescence quenching in a coacervate droplet. These experiments were performed in a much smaller region than the acoustic patterning studies as heme was directly introduced into a droplet of the coacervate suspension containing *apo*-mAPXmEGFP. This prevents any time lag associated with heme diffusion across the sample.

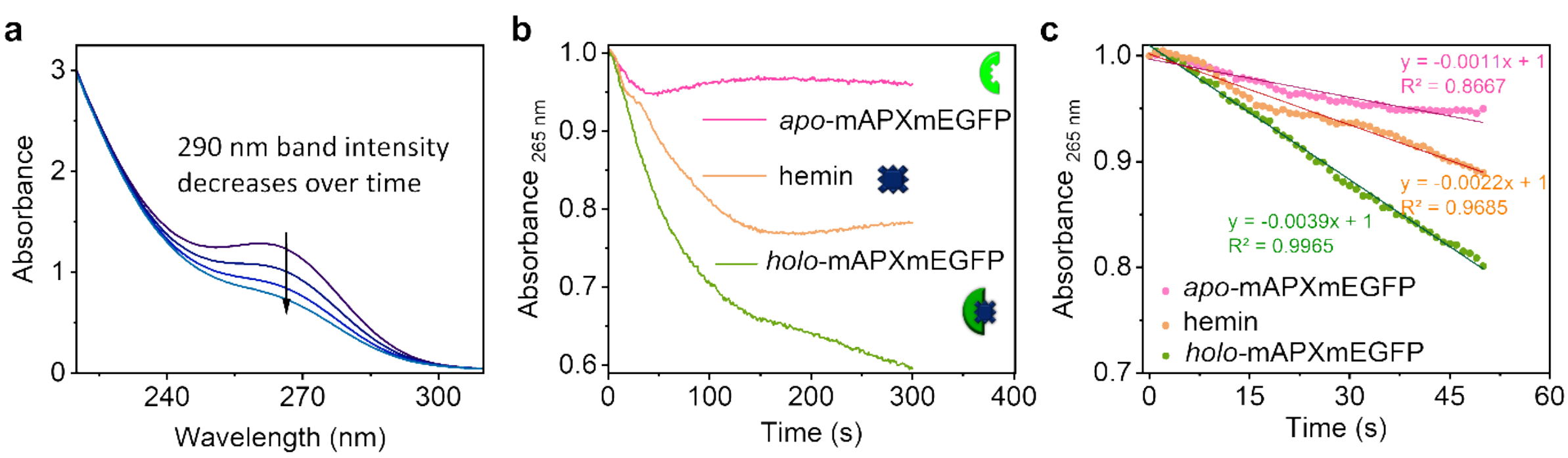

Supplementary Fig. S5: Spectroscopic monitoring of peroxidase activities. a) Absorbance changes over time showing the consumption of ascorbate to generate monodehydroascorbate (MDA) observed from the decrease

of 290 nm band. b) Time-dependent absorption changes monitored at 265 nm for *apo*-mAPXmEGFP (pink), heme (orange) and *holo*-mAPXmEGFP (green). c) Peroxidase activity estimated from initial linear decays obtained from b.[5]

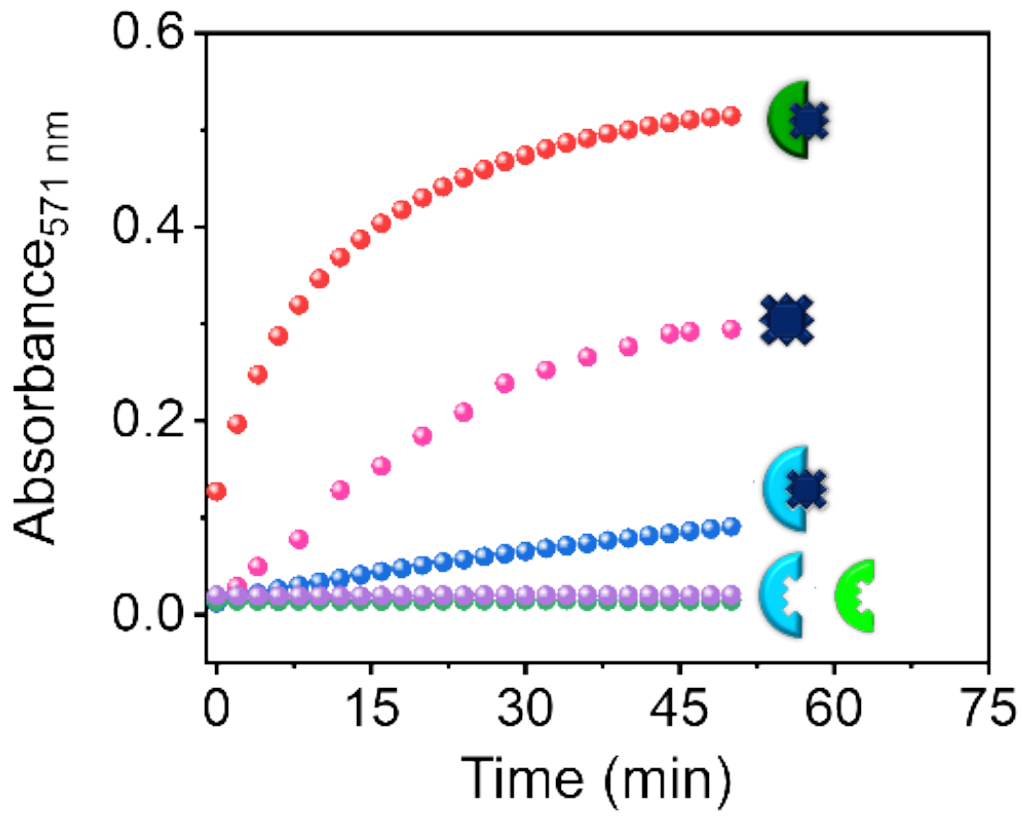

Supplementary Fig. S6: Comparative peroxidase activities of proteins in their *apo* and *holo* states. Absorption changes showing the conversion of amplex red to resorufin in presence of *holo*-mAPXmEGFP (red), heme (pink), *holo*-gMb (blue), *apo*-gMb (purple) and *apo*-mAPXmEGFP (green) with H_2O_2 monitored over time at 571 nm (5 μM of *apo*-mAPXmEGFP, 30 μM heme and 14 μM *apo*-gMb). Negligible resorufin formation was observed in the presence of *apo*-Mb, confirming the absence of intrinsic peroxidase activity. Similarly, free heme exhibited substantially lower peroxidase activity than *holo*-mAPXmEGFP, with no appreciable resorufin generation detected over the 50 min observation period.

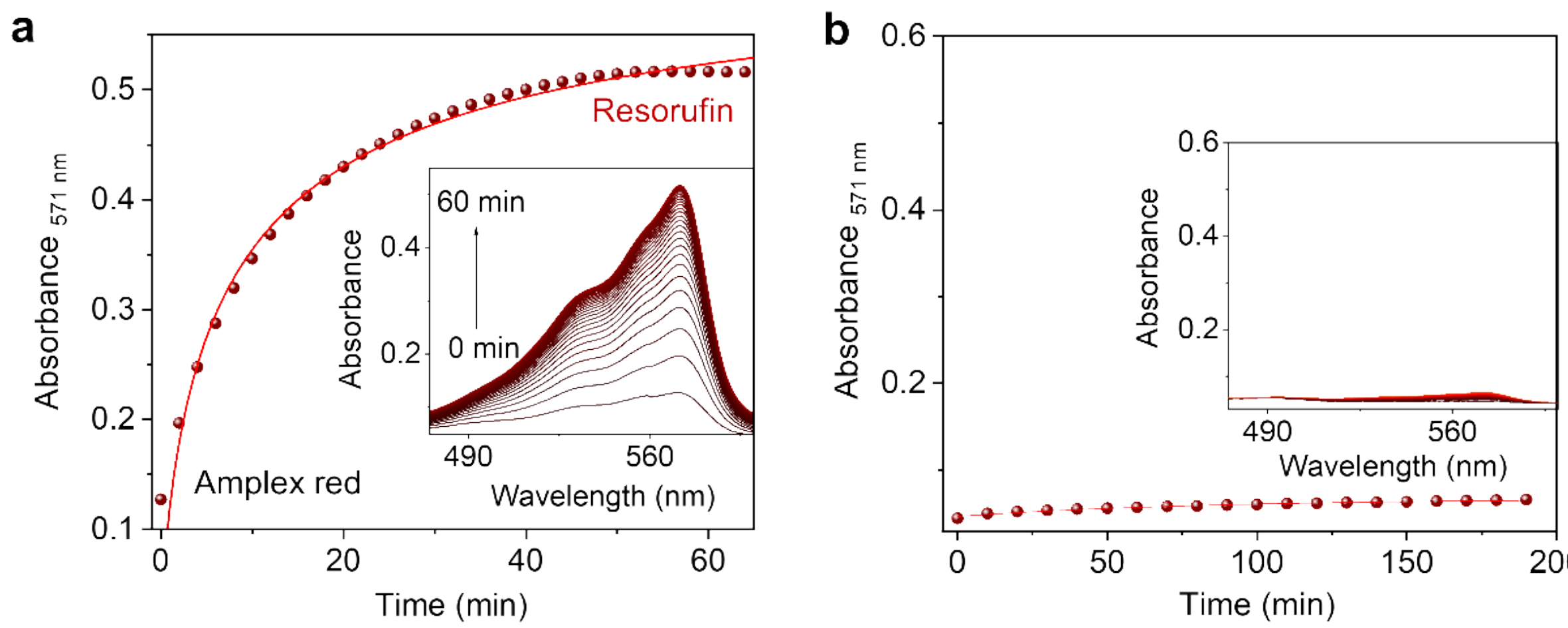

Supplementary Fig. S7: Activity of *holo*- and *apo*-mAPXmEGFP with amplex red in solution. Absorption changes showing the conversion of amplex red to resorufin in presence of a) *holo*-mAPXmEGFP and b) *apo*-mAPXmEGFP in presence of H_2O_2 monitored over time at 571 nm. Negligible resorufin generation is observed for *apo*-mAPXmEGFP, showing the absence of any peroxidase activities.

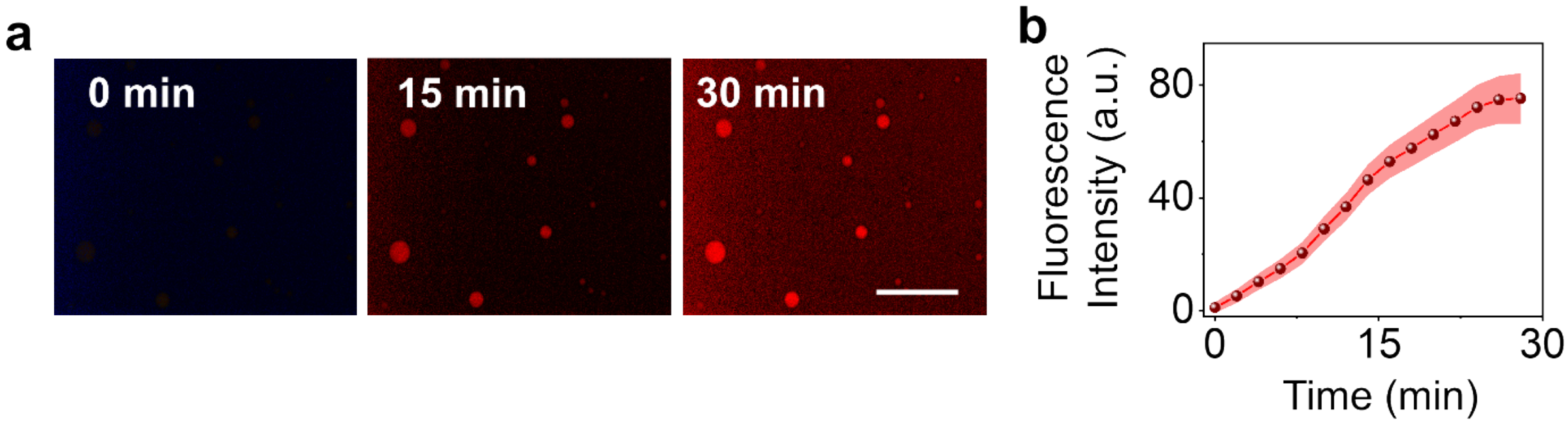

Supplementary Fig. S8: Activity of *holo*-mAPXmEGFP in PDDA/ATP coacervate droplets. a) Time lapse fluorescence microscopy images and b) time-dependent fluorescence intensity changes for *holo*-mAPXmEGFP-containing PDDA/ATP coacervate microdroplets. In the presence of H_2O_2, non-fluorescent Amplex red is converted to fluorescent resorufin. λ$_{ex}$ = 515 - 560 nm and λ$_{em\ cutoff}$ = 580 nm. Scale bar- 50 μm.

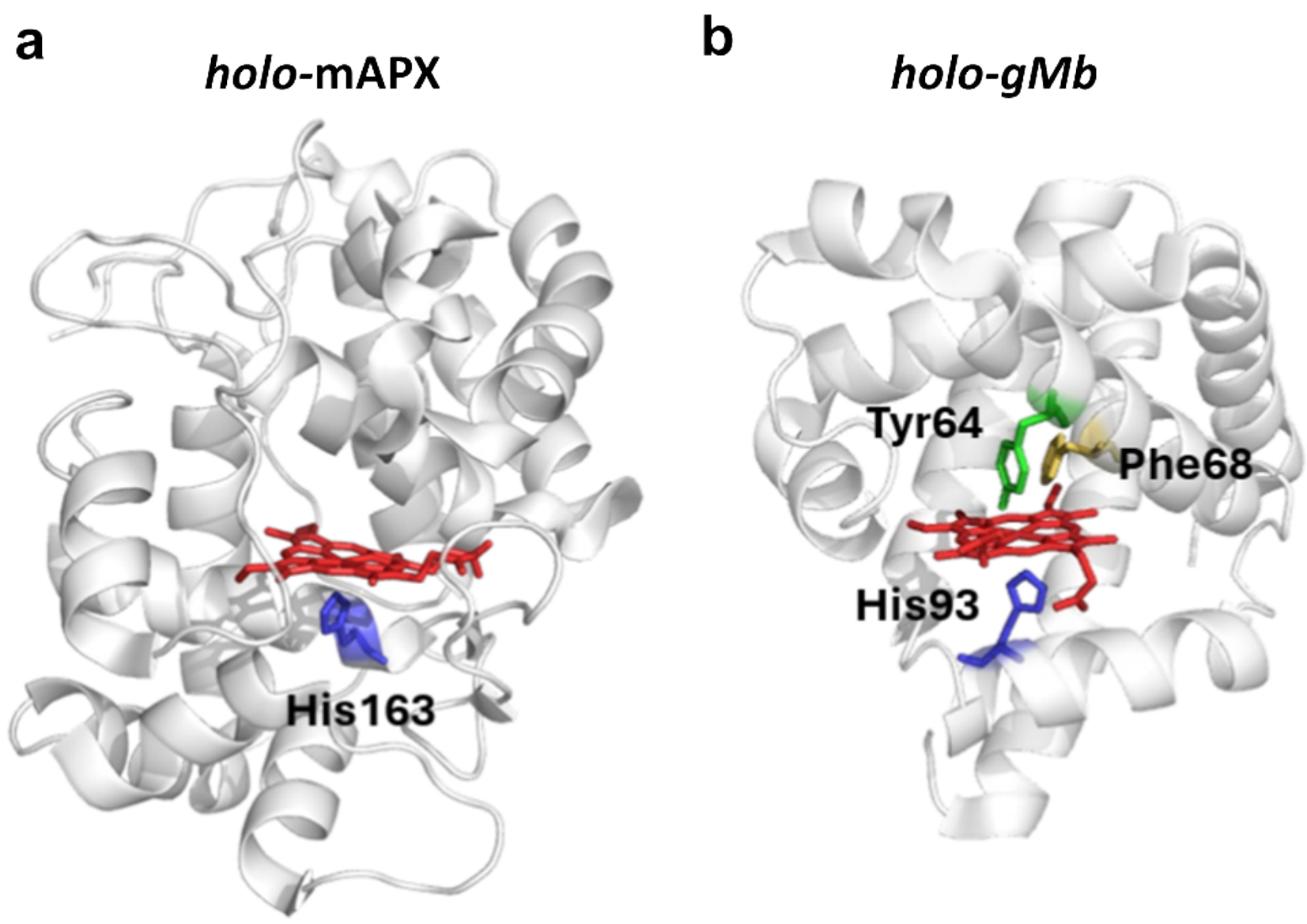

Supplementary Fig. S9: Crystal structures of *holo*-mAPX[6] and *holo*-gMb.[7] a) His163 shown in blue binds is the axial ligand to the heme in ascorbate peroxidase (PDB ID: 1OAG)[8]. b) Tyr64 (green), Phe68 (yellow), and His93 (blue) are shown for *holo*-gMb[9] (H64Y/V68F variant). The structure of gMb was mutated in PyMol, using the crystal structure of the wild type sperm whale protein (PDB ID: 1JP6).

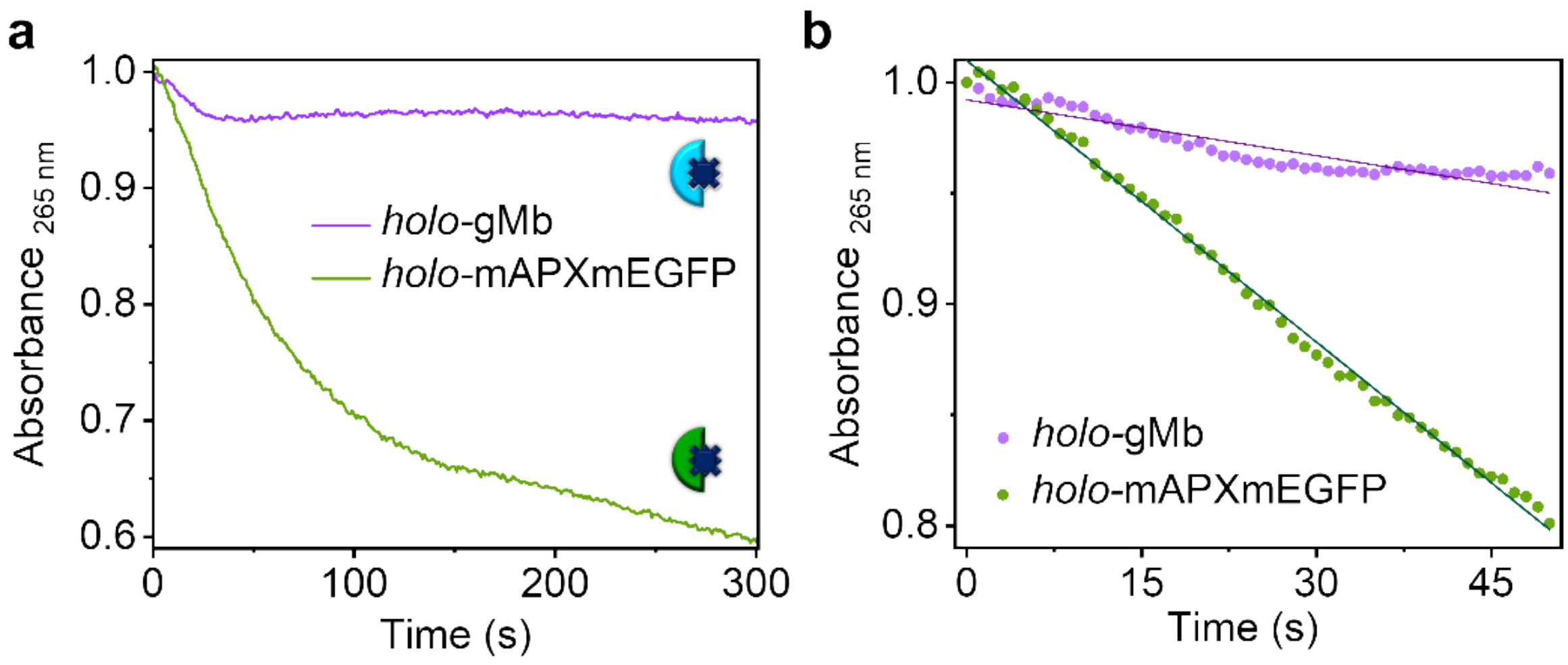

Supplementary Fig. S10: Activity of *holo*-mAPXmEGFP and *holo*-gMb with ascorbate in solution. a) Absorbance changes over time showing the consumption of ascorbate to generate monodehydroascorbate (MDA) monitored at 265 nm for *holo*-gMb (purple) and *holo*-mAPXmEGFP (green). b) Peroxidase activity is estimated from initial linear decays obtained from a.

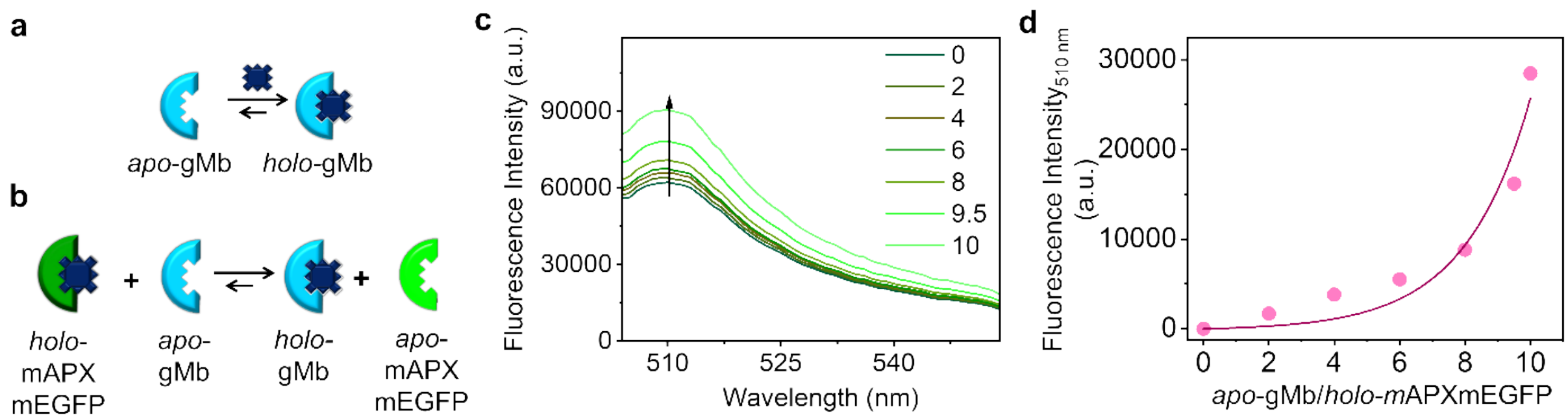

Supplementary Fig. S11: Heme scavenging by *apo*-gMb. Schematic representation of a) heme binding to *apo*-gMb giving *holo*-gMb and b) competitive binding and scavenging of heme by *apo*-gMb from *holo*-mAPXmEGFP showing the eventual recovery of *apo*-mAPXmEGFP fluorescence. c) Fluorescence titration spectra and d) fluorescence changes of *holo*-mAPXmEGFP monitored at 510 nm by increasing concentrations of *apo*-gMb showing the scavenging of heme resulting in the formation of *apo*-mAPXmEGFP. The increase in fluorescence shows the stepwise removal of heme from *holo*-mAPXmEGFP to bind with *holo*-gMb. Increasing the apo-gMb/holo-APX ratio enhances the driving force for heme transfer from holo-APX to apo-gMb. Accordingly, high apo-gMb/holo-hemoprotein ratios (up to 10) are typically used for this assay.[10]

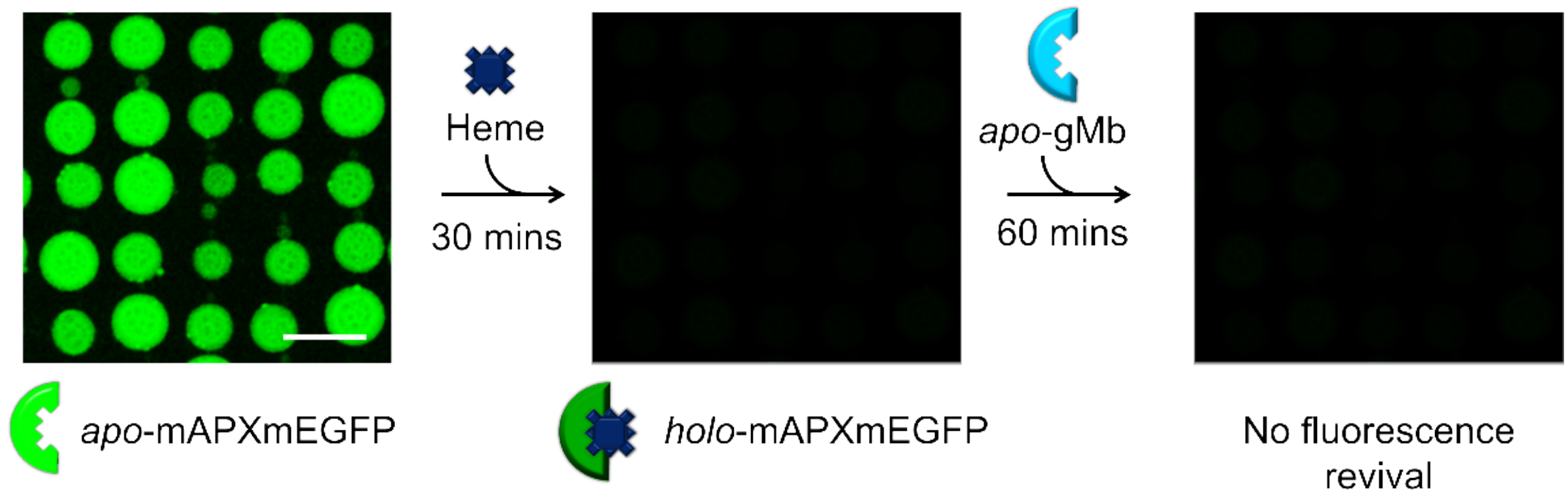

Supplementary Fig. S12: Absence of heme scavenging in coacervate droplets. Sequential fluorescence microscopy images of a 5x5 grid of acoustically patterned PDDA/ATP coacervate droplets containing *apo*-mAPXmEGF. Introduction of heme resulted in fluorescence quenching to generate non-fluorescent *holo*-mAPXmEGFP within the PDDA/ATP coacervate droplets after 30 mins. Further addition of *apo*-gMb into the solution did not restore the green fluorescence, indicating the absence of protein-protein scavenging inside the coacervate droplets.

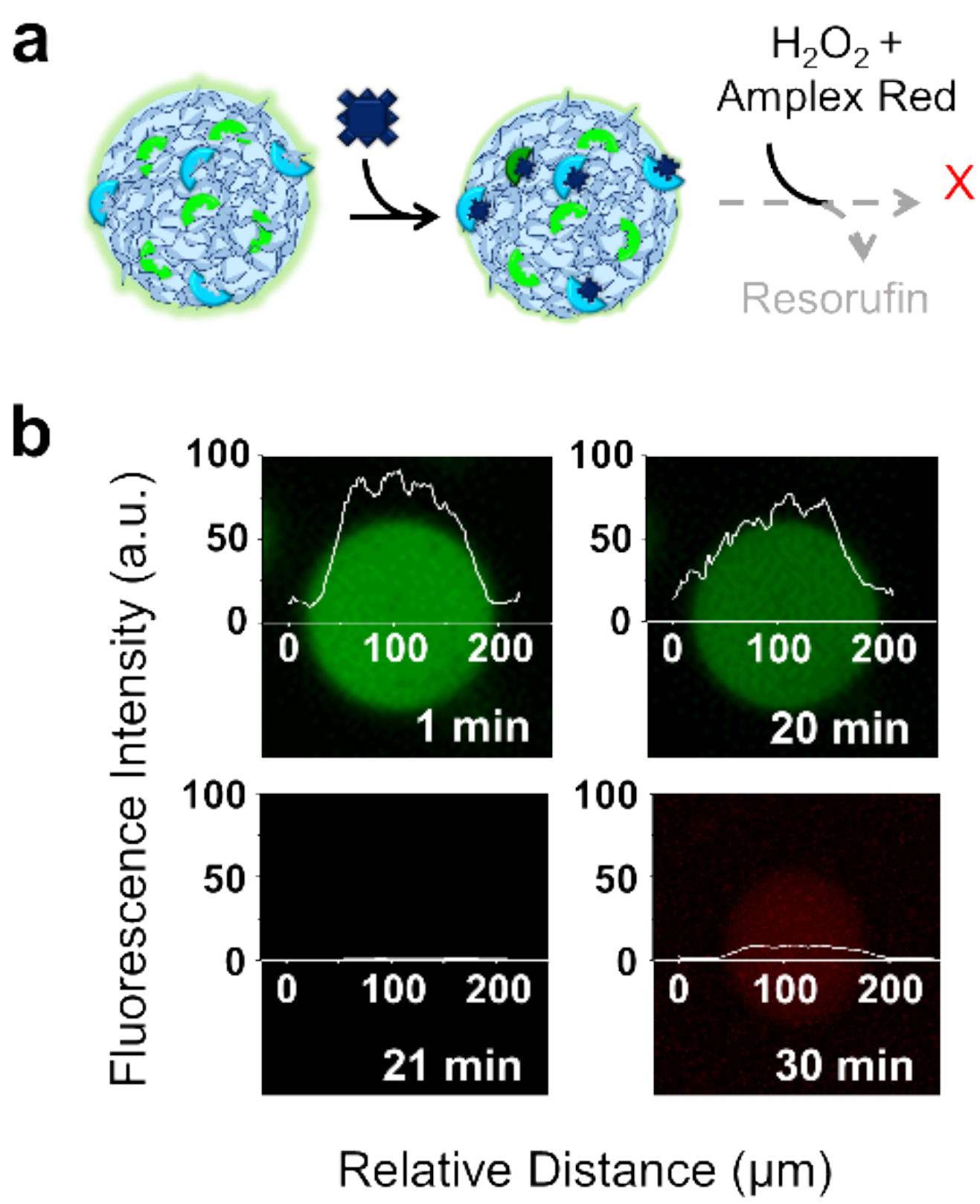

Supplementary Fig. S13: Preferential binding of heme to *apo*-gMb. a) Schematic representation of competitive heme binding between *apo*-mAPXmEGFP and *apo*-gMb where heme preferentially binds to *apo*-gMb to produce *holo*-gMb when encapsulated in PDDA/ATP coacervate microdroplets. b) Fluorescence microscopy images of PDDA/ATP condensate droplets showing inhibited *apo*-mAPXmEGFP

activation in the presence of co-encapsulated *apo*-gMb. As a result, subdued Amplex red conversion to resorufin in the presence of H_2O_2 occurs. Insets show the fluorescence intensity changes of *apo*-mAPXmEGFP encapsulated in PDDA/ATP coacervate droplets (top) and negligible increase in resorufin fluorescence (bottom). The fluorescence images are magnified views of single coacervate microdroplets recorded from a 2D microdroplet array.

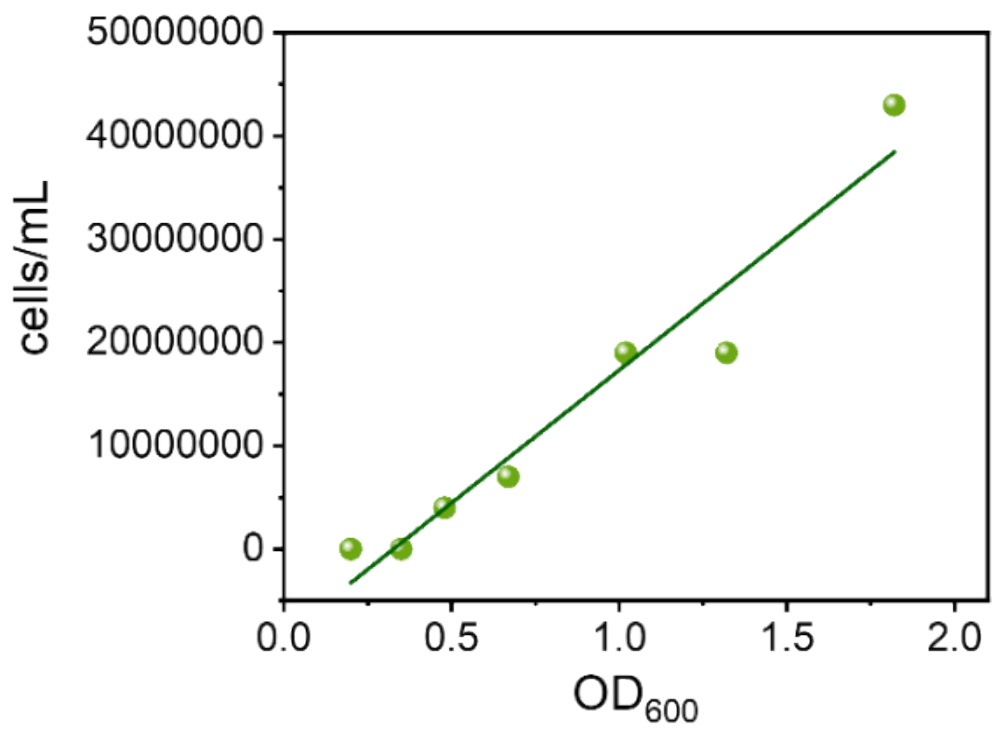

Supplementary Fig. S14: Calibration curve of cell numbers and optical density at 600 nm. An OD_{600} value of 1.0 equates to 3 x 10^7 cells/mL. Data calculated from Colony Forming Unit(s) by diluting the culture sequentially.

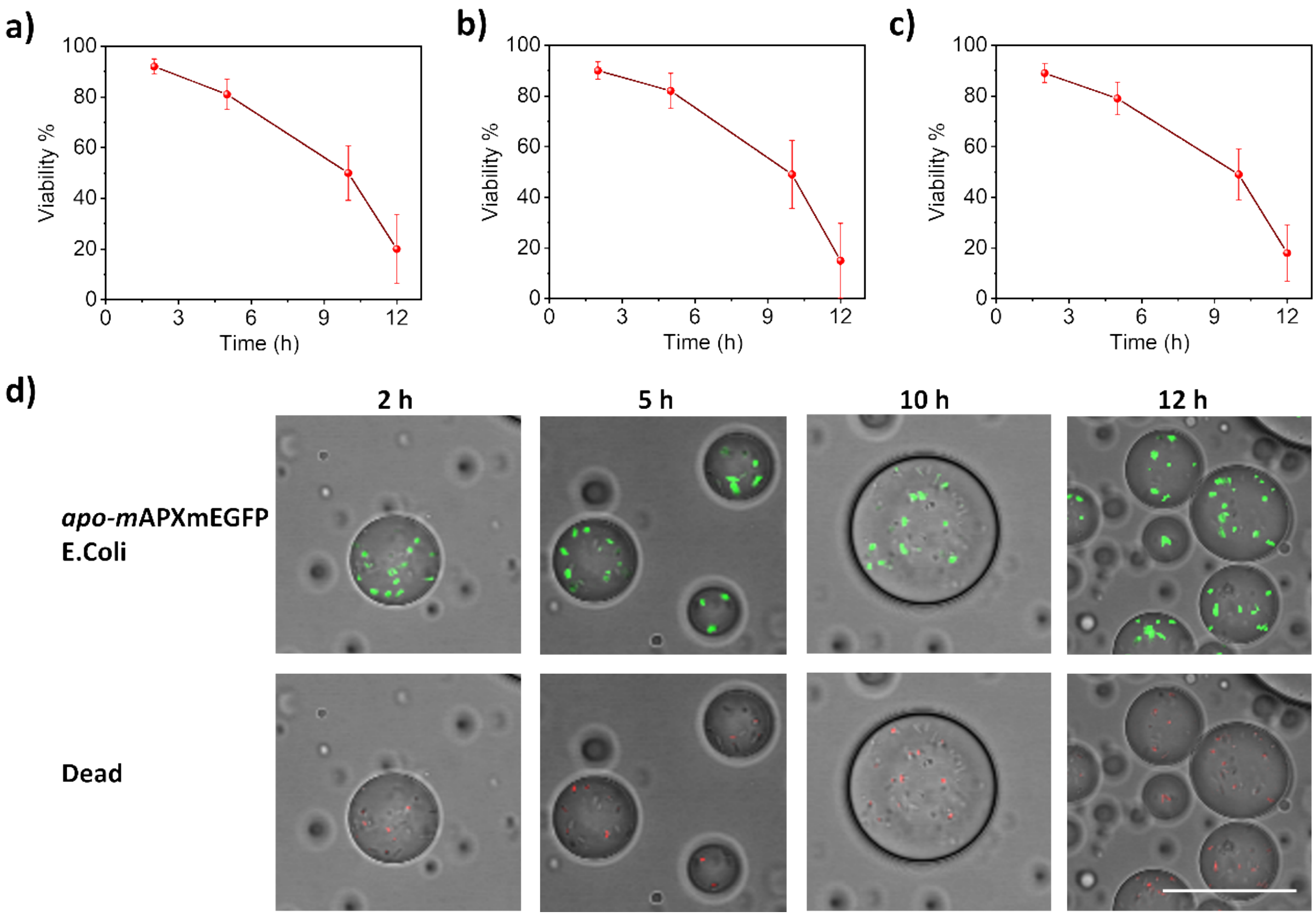

Supplementary Fig. S15: Viability experiments on *apo*-mAPXmEGFP-*E.coli* loaded coacervate droplets. Viability % of *apo*-mAPXmEGFP-*E.coli* cells when trapped in a) PDDA/ATP coacervate microdroplets, b)

E.coli containing PDDA/ATP coacervate droplets in the presence of 30 μM heme, and c) *E.coli* containing PDDA/ATP coacervates in the presence of 0.54 mM H_2O_2 and 0.22 mM Amplex Red. d) Representative overlaid brightfield and confocal fluorescence microscopy images of propidium iodide-treated *apo*-mAPXmEGFP-*E.coli*-loaded condensate droplets at different times. Scale bar, 50 μm. 80% of the *E. coli* cells are alive when encapsulated in the PDDA/ATP matrix for at least 5 h, after which cell viability decreases. At each time point, a sample size of n = 100 coacervate droplets was analysed, and the percentages of live and dead *E. coli* cells were determined accordingly.

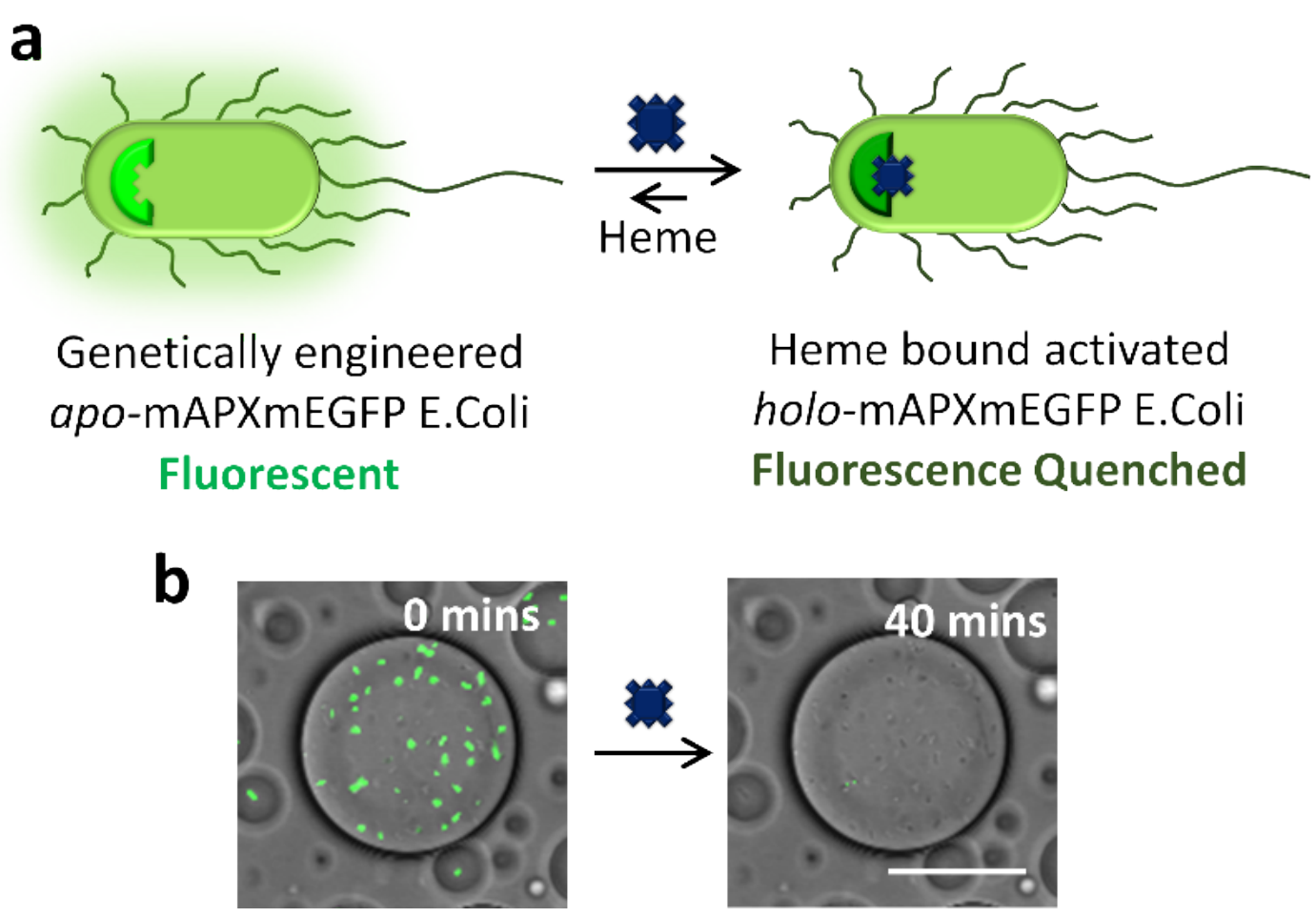

Supplementary Fig. S16: Heme-induced fluorescence quenching of *apo*-mAPXmEGFP-*E.coli* cells in freely suspended coacervate droplets. a) Schematic representation of fluorescence quenching of *apo*-mAPXmEGFP-*E.coli* by heme. b) Confocal microscopy images showing heme-induced fluorescence quenching of *apo*-mAPXmEGFP-*E.coli* within individual PDDA/ATP coacervate microdroplets over 20 mins. Scale bar, 50 μm.

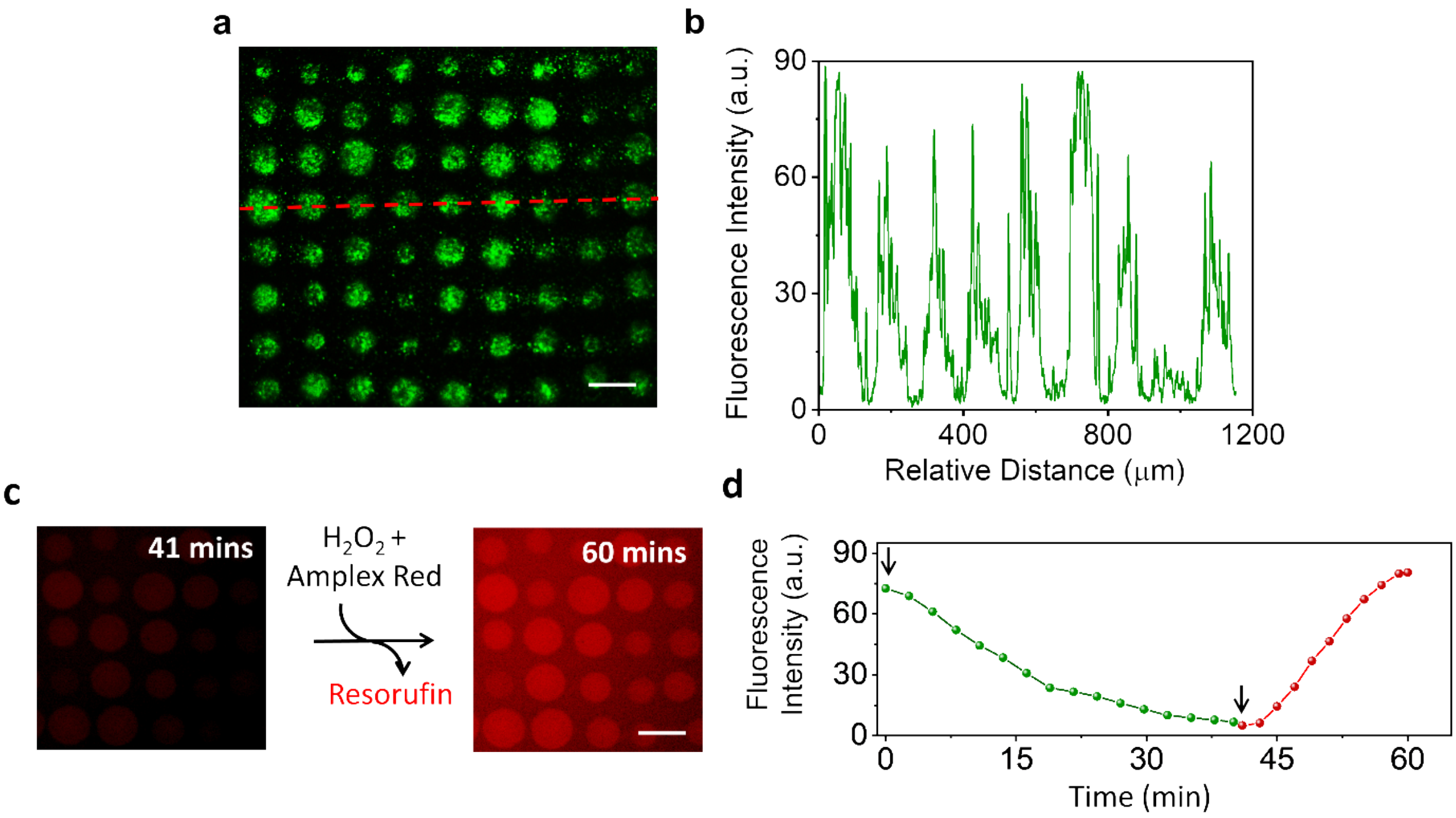

Supplementary Fig. S17: Heme-induced fluorescence quenching and reactivity of *apo*-mAPXmEGFP-*E.coli* cells in a 2D microarray of coacervate droplets. a) Fluorescence microscopy images of acoustically patterned PDDA/ATP coacervate droplets containing *apo*-mAPXmEGFP-*E.coli* cells. b) Fluorescence trace across the dashed red line shown in a. c) Red fluorescence evolution showing the formation of resorufin from Amplex red in the presence of H_2O_2 catalyzed by *holo*-mAPXmEGFP-*E.coli* cells present within the coacervate droplets. Scale bar, 150 μm. d) Temporal fluorescence changes showing heme-induced fluorescence quenching of *apo*-mAPXmEGFP-*E.coli* cells and concomitant increase in peroxidase activity.

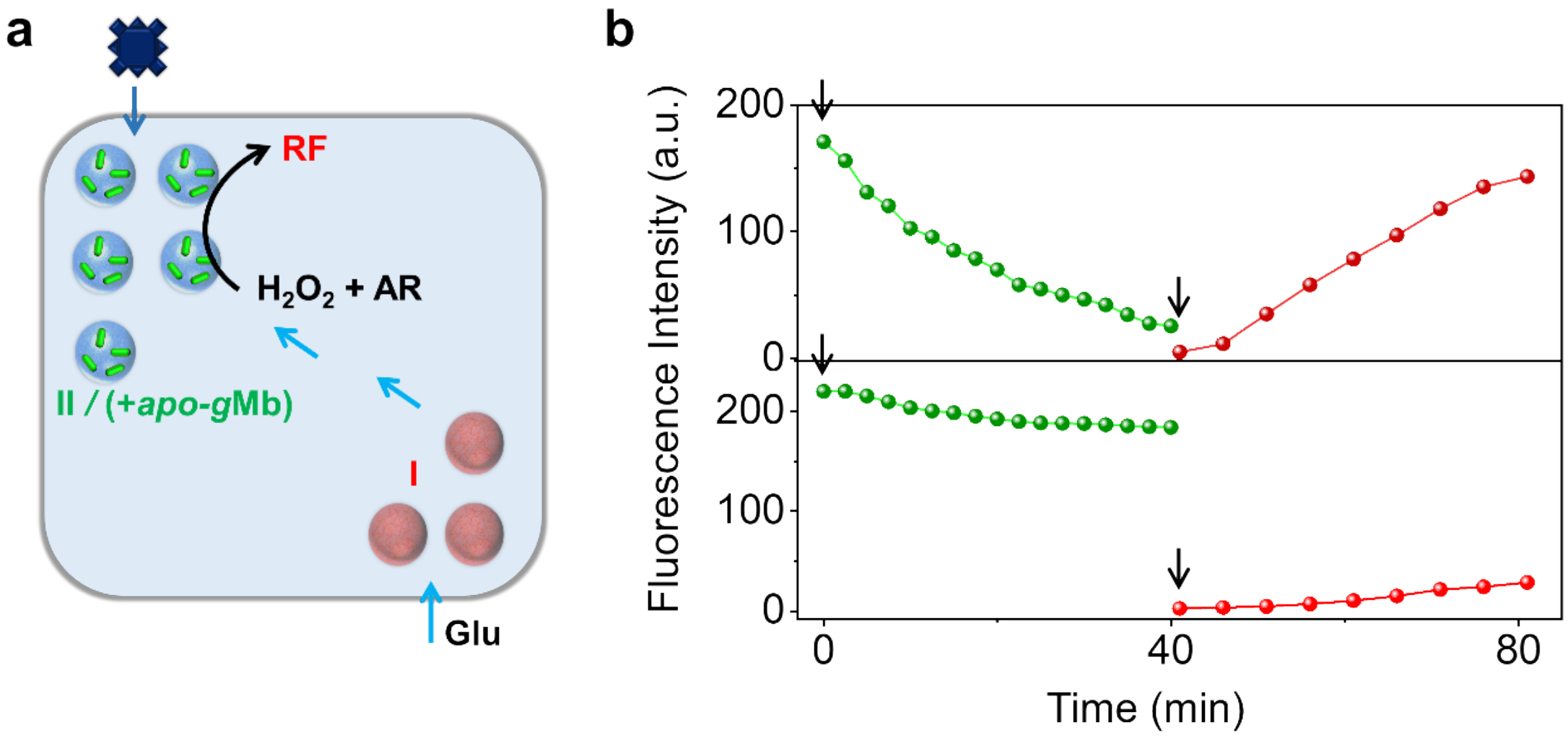

Supplementary Fig. S18: Heme-mediated chemical signaling in synthetic/living condensate droplet networks. a) Schematic representation of a spatially distributed binary population of PDDA/ATP coacervate droplets containing two different biocatalytic modules. Module **I**, RITC-GOx (red fluorescence, YES gate, signal generator); Module **II**, *apo*-mAPXmEGFP-*E.coli* cells (green fluorescence) without or with *apo*-gMb (AND gate, signal effector). Glucose (A, Glu), heme (B, blue symbol), Amplex red (AR), resorufin

fluorescence (RF), hydrogen peroxide (H_2O_2). b) Fluorescence changes over time obtained from module **II** for heme-induced fluorescence quenching for *apo*-mAPXmEGFP-*E.coli* (monitored for 40 mins) and subsequent peroxidase-induced transformation of Amplex red to resorufin in the presence of H_2O_2 (monitored for 40 mins). A decrease in green fluorescence (*apo*-mAPXmEGFP activation to *holo*-mAPXmEGFP by heme binding) is observed, which subsequently catalyzes Amplex red conversion due to communication between the modules (top panel). These changes are inhibited if *apo*-gMb is co-encapsulated with *apo*-mAPXmEGFP-*E.coli* cells in Module 3 (bottom panel).

3. Supplementary Tables

[catalyst] = 116 μM	Peroxidase activity units (s^{-1})	%
apo-mAPXmEGFP	80	33
heme	135	56
*holo-m*APXmEGFP	240	100
holo-gMb	68	28

Supplementary Table S1. Calculated peroxidase activity values.[5,11,12]

Heme-protein interaction	Dissociation Constant K_d
apo-gMb	10^{-11} M
holo-mAPXmEGFP	22×10^{-9} M

Supplementary Table S2. Experimental K_d values for proteins with heme.

Encapsulated component	Total Concentration	Partition coefficient
heme	30 μM	5.62
apo-gMb	14 μM	3.21
apo-mAPXmEGFP	5 μM	2.72

Supplementary Table S3. Partition coefficients of the encapsulated components inside PDDA/ATP coacervates at pH 8.

4. Supplementary References

1. G. C.-H. Leung, S. S.-P. Fung, A. E. Gallio, R. Blore, D. Alibhai, E. L. Raven and A. J. Hudson. *Proc. Natl. Acad. Sci. U.S.A.* 2021, **118**, e2104008118.
2. A. E. Gallio, N. A. Marson, K. J. Heesom, P. A. Lewis, D. Alibhai, C. A. Dugdale, A. Hermand, J. Basrane, A. J. Hudsone and E. L. Raven. *Proc. Natl. Acad. Sci. U S A*, 2025, 122, e2508237122.
3. E. Sitte, M. O. Senge. *Eur. J. Org. Chem.* 2020, 3171-3191.

4. F. W. J. Teale. *Biochimica et Biophysica Acta* 1959, **35**, 543.

5. R. Mittler and B. A. Zilinskas. *Plant Physiol.* 1991, **97**, 962-968.

6. RCSB PDB - 1OAG: Ascorbate peroxidase from soybean cytosol.

7. RCSB PDB - 1MGN: HIS64(E7)□ TYR Apomyoglobin as a reagent for measuring rates of hemin dissociation.

8. Katherine H. Sharp, Martin Mewies, Peter C.E. Moody & Emma Lloyd Raven. *Nat. Struct. Mol. Biol.* 2003, **10**, 303-307.

9. RCSB PDB - 1MGN: HIS64(E7)□ TYR Apomyoglobin as a reagent for measuring rates of hemin dissociation.

10. M. T. Nelp, P. A. Kates, J. T. Hunt, J. A. Newitt, A. Balog, D. Maley, X. Zhu, L. Abell, A. Allentoff, R. Borzilleri, H. A. Lewis, Z. Lin, S. P. Seitz, C. Yan and J. T. Groves. *Proc. Natl. Acad. Sci. U.S.A.* 2018, **115**, 3249-3254.

11. I. K. Macdonald, S. K. Badyal, L. Ghamsari, P. C. E. Moody and E. L. Raven. *Biochemistry* 2006, **45**, 7808-7817

12. L. Lad, M. Mewies and E. L. Raven. *Biochemistry* 2002, **41**, 13774-13781.
